# Neuroinflammation in Parkinson’s Disease – Putative Pathomechanisms and Targets for Disease-Modification

**DOI:** 10.3389/fimmu.2022.878771

**Published:** 2022-05-18

**Authors:** Alexander Grotemeyer, Rhonda Leah McFleder, Jingjing Wu, Jörg Wischhusen, Chi Wang Ip

**Affiliations:** ^1^Department of Neurology, University Hospital Würzburg, Würzburg, Germany; ^2^Section for Experimental Tumor Immunology, Department of Obstetrics and Gynecology, University Hospital of Würzburg, Würzburg, Germany

**Keywords:** Parkinson’s disease, neuroinflammation, T cells, microglia, neurodegeneration, animal models, inflammatory cascades

## Abstract

Parkinson’s disease (PD) is a progressive and debilitating chronic disease that affects more than six million people worldwide, with rising prevalence. The hallmarks of PD are motor deficits, the spreading of pathological α-synuclein clusters in the central nervous system, and neuroinflammatory processes. PD is treated symptomatically, as no causally-acting drug or procedure has been successfully established for clinical use. Various pathways contributing to dopaminergic neuron loss in PD have been investigated and described to interact with the innate and adaptive immune system. We discuss the possible contribution of interconnected pathways related to the immune response, focusing on the pathophysiology and neurodegeneration of PD. In addition, we provide an overview of clinical trials targeting neuroinflammation in PD.

## 1 Introduction

Parkinson’s disease (PD) is the second most common neurodegenerative disease after Alzheimer’s disease (AD) (1–4). Motor symptoms such as bradykinesia, rigidity, resting tremor, and postural instability (5, 6) are hallmarks of PD and essential for staging. PD patients also exhibit a wide variety of non-motor symptoms, ranging from anosmia, rapid eye movement (REM) sleep disorders, and constipation to severe psychiatric symptoms such as dementia (6). Some of these non-motor symptoms are observed long before motor counterparts occur and are therefore essential to early PD diagnosis (7). From the defined histological description, a staging model arose in which progression of PD symptoms can be matched with intracellular deposits of pathological α-synuclein aggregates (pαSYN) or Lewy pathology (LP) of the corresponding brain areas (8–11). However, LP is also observed in the brains of older patients with no PD symptoms during their lifetime, indicating that LP is not specific as a standalone for PD diagnosis (10, 12). Moreover, it remains unclear why pαSYN occurs, why pαSYN spreads from cell to cell (10, 13–16), and how cell death is mediated by pαSYN (17). Since α-synuclein (αSYN) has been described as a critical mediator of inflammation and immune responses, and is released from inflamed neurons, αSYN may trigger a self-amplifying proinflammatory response (18). Intriguingly, neuroinflammation has emerged as a key aspect in PD (19–22) – but the extent to which neuroinflammation contributes to the development and maintenance of PD is controversial. Hence, knowledge transfer from neuroinflammatory diseases, where the concept of immune system-induced neuronal cell damage is established, could help to elucidate the still-obscure role of the immune system in PD.

Immunomodulatory therapies are established as effective treatments in ‘classical’ inflammatory neurological diseases, such as multiple sclerosis (MS), neuromyelitis optica spectrum disorders (NMOSD), or myasthenia gravis (MG) (23–27). Although the pathophysiology differs regarding the antigen in NMOSD and MG, common therapeutic strategies are implemented (28). In both entities, antibodies are pathophysiologically relevant and inhibition of the complement system has proven beneficial (29, 30). This supports the idea that similar inflammatory processes can be addressed across different neuroinflammatory diseases.

Various concepts have been tested as a causal treatment for PD, including vaccination against αSYN (31, 32). However, none have yet proven successful and traditional treatment of PD is still symptomatic and largely based on dopaminergic medication (33–35). To determine the relevance of neuroinflammation in PD pathogenesis, large-scale clinical observational studies are essential to generate appropriate hypotheses, which can be subsequently tested in animal models. In our review, we discuss the evidence for neuroinflammation in PD and potential future targets. We further illustrate immunological pathways linked to neuroinflammation and dopaminergic neurodegeneration.

## 2 Pathomechanisms of Neuroinflammation in PD Patients

The pathophysiologic role of the immune system in PD is still enigmatic. Neuroinflammation was proposed by James Parkinson himself in the initial description of PD (1). There is also early literature discussing PD as an ‘autoimmune disease’ (36). Yet interest in the topic was only rekindled in 1988, when microglia activation was described in brain autopsies of PD and AD patients (21). Since then, evidence has accumulated that neuroinflammation is likely to play a critical role in PD (36–38). Significant elevation of inflammatory cytokines in the blood and cerebrospinal fluid (CSF) of PD patients has now been confirmed in a meta-analysis over 25 clinical studies (39). Increased expression of NLRP3 and caspase-1 genes in peripheral blood mononuclear cells (PBMC) and elevated protein levels of NLRP3, caspase-1, and IL-1B in the blood plasma were found to correlate with PD severity (40), further supporting the perception of PD as a chronic systemic inflammatory disease. Evidence suggests that both innate and adaptive immune responses are involved in PD progression (41–44).

### 2.1 Innate Immune Response

Microglia are permanently in contact with astrocytes, neurons, and endothelial cells, constantly monitoring surrounding tissue for an ongoing infection or trauma *via* thin processes (45). In response to injury or infection, resting microglia undergo morphological alterations, change their transcriptional activity, and present antigens *via* the major histocompatibility complexes (MHC)-I and -II (46–48). Unlike MHC-I, which is constitutively expressed on all nucleated cells and platelets (49–52), MHC-II is specifically expressed on antigen-presenting cells such as microglia, astrocytes, monocytes, macrophages, dendritic cells, and granulocytes, and can be induced upon activation.

As brain-resident innate immune cells, microglia are typically the first responders to altered homeostasis within the CNS. Microglia activation increases the amount of nuclear factor ‘kappa-light-chain-enhancer’ of activated B-cells (NFKB) and NLR family pyrin domain-containing 3 (NLRP3), with subsequent upregulation of nicotinamide adenine dinucleotide phosphate (NADPH)-oxidase and cytokines such as interleukin-1 beta (IL-1B) and tumor necrosis factor alpha (TNF) (53, 54). Phagocytic activity and MHC-II expression are also enhanced (55, 56). An increase in activated microglia has been observed in both PD patients and healthy older people, indicating that various triggers might induce a proinflammatory shift with age (21, 57). Compared to age-matched controls, PD patients exhibited significantly elevated levels of proinflammatory cytokines such as IL-1B, IL-6, and TNF in CSF and blood (39, 58). Furthermore, ([^11^C](*R*)-PK11195)-based positron emission tomography (PET) has revealed a higher density of activated microglia in the midbrain and putamen of early PD patients (59, 60), which correlated with decreased activity of dopamine transporter ligands ([^11^C] CFT). Similar results were described in patients suffering from a REM sleep behavior disorder (60, 61). While the presence of chronic inflammatory processes in PD is now widely accepted, the underlying reason for neuroinflammation is still unclear. Due to its function as a damage-associated molecular pattern (DAMP) (53, 62, 63), pαSYN might trigger and maintain a proinflammatory shift of the immune system. In addition, DAMPs may be released from dying or damaged cells. Well-known DAMPs triggering an innate immune response upon interaction with pattern recognition receptors (PRRs) are IL-1α or mitochondrial reactive oxygen species (mROS), which activate NLRP3. Consecutive NLRP3 activation leads to increased IL-1B synthesis as a trigger for further innate immune responses (64). Initial microglia activation in PD (21, 65) may therefore result from PRR-mediated responses to DAMPs.

In addition to DAMPs, pathogen-associated molecular patterns such as viral RNA or the bacterial cell wall component lipopolysaccharide (LPS) may also maintain neuroinflammation. While a correlation between viral infection and PD has not strictly been established, preceding infections might still modulate the risk for developing PD (66). For example, there is evidence for toxin-related PD, which implies that toxins released by infectious agents might also be responsible for some cases of ‘sporadic’ PD. Other toxins such as the pesticide rotenone have neurotoxic and neuroinflammatory effects that may kill dopaminergic cells and thereby cause PD in people exposed to higher concentrations (67).

Possible links between PD and infection have become even more topical with the still-ongoing worldwide COVID-19 pandemic. While there is very little consolidated knowledge regarding possible interactions between the severe acute respiratory syndrome coronavirus 2 (SARS-CoV-2) and PD, there are some interesting observations on pathogenic coronaviruses and neuroinflammation or, more specifically, PD. Antibodies against coronavirus antigens have been found in the CSF of PD patients (68). In addition, intranasal infection of hACE2 transgenic mice with SARS-CoV-1 caused severe CNS infections that spread from the olfactory bulb to the basal ganglia and midbrain within approximately four days (69). Considering that COVID-19 patients often experience anosmia, SARS-CoV-2 is also likely to reach the CNS *via* the olfactory bulb. Disconcertingly, *in vitro* assays have shown that the nucleocapsid(N)-protein of SARS-CoV-2 accelerates pαSyn-aggregation by a factor of 10 (70). Thus, it is concerning that COVID-19-related inflammation may trigger or accelerate PD in patients at risk. Yet to date, there are only a few case studies describing the development of PD after SARS-CoV-2 infection (71, 72). Whether infection with SARS-CoV-2 impacts the development of PD remains to be explored.

### 2.2 Adaptive Immune Response in PD: T Cells Friends and Foes

Neuroinflammation in PD is not confined to the innate immune system; it also involves adaptive immune responses. Several findings indicate that various T cell subpopulations may contribute to PD pathophysiology (73).

Neuroinflammation within the substantia nigra (SN) is well-described in PD. Significant infiltration of both CD4^+^ and CD8^+^ T cells into the SN of PD patients has been described (42), particularly elevated levels of CD8^+^ T cells (74, 75). It is possible that CD8^+^ T cells have an important role in early PD, even before pαSYN can be detected in the SN, as relevant infiltration into the SN has been observed in very early PD that subsides with disease progression (74). Altered counts of CD4^+^ T cell populations in the blood of PD patients have also been reported, but remain controversial. Many studies describe a decrease in the overall CD4^+^ T cell population in PD patients (44, 76–78). However, both elevated (76) and reduced T_reg_ cell counts (79) were found in the blood of PD patients, although the studies used a relatively non-specific marker (CD4^+^CD25^+^) to identify T_reg_ cells. Increased numbers of CD4^+^FoxP3^+^ T_reg_ have been observed in the blood of PD (aged 46-80 years) and AD (62-82 years) patients and healthy older people (51-87 years) compared to healthy young controls (23-40 years) (80). An increase in T_reg_ cell activity with age was also seen, which was significantly more pronounced in PD and AD patients compared to young controls (80). In contrast, decreased levels of CD4^+^CD25^high^CD127^low^ T_reg_ cells (44, 81) and reduced suppressive activity of T_reg_ cells has also been found in PD patients (77). Furthermore, peripherally-reduced T_reg_ cells and increased Th1 cell counts were found to correlate with the severity of motor dysfunction (assessed by Unified Parkinson’s Disease Rating Scale III [UPDRS III]) (44, 81). In addition, decreased Th2 cell numbers and a relative increase in Th1 cells were seen in PD patients (44). Despite these discrepancies, most studies highlight an increased peripheral immune response in PD, with a reduced amount of T_reg_ cells (CD4^+^CD25^high^CD127^low^) (44, 81) (**Table 1**).

**Table 1:** Overview of human and animal studies on T cells with special regard to T_reg_ cells.

*Publication*	*derived from*	*origin*	*Major finding (referring to PD)*	*T_reg_ identification (total amount)*
(42), *2009*	brain tissue	human, mouse	sign. infiltration of CD4^+^ and CD8^+^ T cells in the SN	-
(74), *2020*	brain tissue	human	sign. infiltration of CD8^+^ T cells in early stages of PD	-
(75), *2022*	brain tissue, spleen	human (brain), mouse (brain, spleen, lymph nodes)	sign. infiltration of CD8^+^ T cells in the SN of humans and of CD4^+^ and CD8^+^ T cells in the SN of AAV1/2-A53T-αSyn mice	-
(76), *2001*	mesenteric lymph nodes	rat	no changes (6-OHDA); increase of CD4^+^CD25^+^ T cells (MPTP)	CD4^+^CD25^+^
(44), *2018*	blood	human	decrease of CD4^+^ T cells, Decrease of Th2, Th17 and T_reg_ cells	CD4^+^CD25^high^CD127^low^
(76), 2001	blood	human	total CD4^+^ T cells decreased, CD4^+^CD25^+^ T cells increased	CD4^+^CD25^+^
(77), *2012*	blood	human	total CD4^+^ T cells decreased.	CD4^+^CD25^high^CD127^low^
			suppressive activity of T_reg_ impaired	
(79), *2005*	blood	human	decrease of T_reg_	CD4^+^CD25^+^
(80), *2007*	blood	human	no change of CD4^+^CD25^+^ T cells but increase in CD4^+^FoxP3^+^ T cells	CD4^+^CD25^high^
				CD4^+^FoxP3^+^
(81), *2015*	blood	human	decrease of CD4^+^, T_reg_ T cells	CD4^+^CD25^high^CD127^low^
			Increase of Th1, Th2, Th17 T cells	

These population and activity shifts of T cells, along with an increase in HLA-DR positive antigen-presenting microglia in PD patients (82), support the idea that CD4^+^ T cells might contribute to neurodegeneration in PD. Studies on the role of Th17 cells in PD have generally confirmed this concept. Addition of IL-17 to autologous cocultures between T cells and pluripotent stem cells (iPSC)-derived mid brain neurons (MBN) from PD patients also induced NFKB-dependent cell death. iPSC-derived MBN from PD patients further showed higher expression of IL-17 receptors, together with enhanced susceptibility towards death induction by recombinant IL-17 or autologous Th17 cells (73). Interestingly, Th17 cells are increased in PD patients, which is consistent with the results of previous studies (81, 83, 84). However, one study in PD patients reported a decrease in the Th17 cell count, along with unchanged levels of IL-17 (44). Based on these studies, Th17 cells and IL-17 are likely to favor the progression of PD, but their distinct role remains unresolved (85, 86). Consequently, a comparison of different analytical approaches might be needed to rule out possible methodological issues.

In addition to flow cytometric analyses, gene expression analyses and genome-wide association studies that investigate alterations in immune response pathways are playing an increasing role in PD research. Expression quantitative trait loci (eQTLs) are obtained by analyzing the genome and transcriptome of patients with correlating alleles and the expression level of transcripts. Based on CD4^+^ T cells and monocytes from a multi-ethnic cohort of 461 healthy individuals, susceptibility alleles for MS, rheumatoid arthritis, type 1 diabetes, and PD were found to be overrepresented in CD4^+^ T cells, while disease- and trait-associated cis-eQTLs associated with AD and PD were primarily overrepresented in monocytes (87). Further evidence for the relevance of the immune system in PD pathogenesis comes from the association of PD with an immune haplotype, including the DRB5*01 and DRB1*15:01 alleles, and from a non-coding polymorphism in the region that might increase MHC-II expression (88). Finally, a very recent study stratified PD patients by their T cell responsiveness to α-SYN as a proxy for an ongoing inflammatory autoimmune response (89). Gene expression analysis in peripheral memory T cells then revealed a clear PD-specific gene signature with transcriptomic markers for inflammation, oxidative stress, phosphorylation, autophagy of mitochondria, cholesterol metabolism, and chemokine signaling *via* CX3CR1, CCR5, and CCR1.

### 2.3 Adaptive Immune Response in PD: B Cells and Humoral Immunity

While T cells are the more prevalent adaptive immune cells in CSF (90), B and T cell immunity are closely intertwined (91). Although immunohistochemical analyses have found B cell-rich tertiary lymphoid structures in most brains from patients with MS, but not in the two PD patients investigated (92), there is ample evidence for the involvement of B cells, antibodies, and humoral immune effector mechanisms in PD (93–95). In a postmortem study of 13 patients with idiopathic PD and three with genetic PD (compared with 12 controls), all PD patients showed IgG (mostly IgG1) but no IgM binding to dopaminergic neurons, while Lewy bodies were strongly immunolabelled with IgG (94). Nearby activated microglia expressed the high-affinity activating IgG receptor FcγRI, which implies a significant role for antibody-dependent cell-mediated cytotoxicity. The inhibitory IgG receptor FcγRII was absent in all cases. Subsequent studies on B cell-related adaptive immune responses in PD support the involvement of B cells and humoral immune effector mechanisms (96–98). Remarkably, some patient sera also contained antibodies capable of neutralizing pαSYN ‘seeding’ (forming of αSYN aggregates) *in vitro* (99). Elevated levels of serum antibodies against αSYN have also been reported in a comparison of sera from PD patients with AD patients and controls (100). Of note, B cell numbers were found to be reduced in PD patients (96, 101), along with an increase in IgA levels (102) that correlated with non-motor symptoms (96). Moreover, cytokine expression of B cells was altered in PD (96). Cytokine-dependent interactions between B cells and Th1 and Th17 cells (103) imply that B cells may also modulate proinflammatory Th17 cells in PD patients (73). In addition, there are relevant interactions between the humoral immune response and the complement system, as described in a comprehensive review (104).

Given the paucity of studies focusing on the distinct function of B cells in PD or other neurodegenerative diseases (103, 105), a more profound understanding of B cell function might reveal new opportunities for disease modification. Again, the targeted transfer of knowledge from ‘classical’ neuroinflammatory disease would be useful (103).

### 2.4 Anti-Inflammatory Disease Modification

Since proinflammatory changes have been consistently observed in PD, several studies analyzed whether suppressing inflammation might modify the disease course of PD. A population-based, case–control study found that patients receiving immunosuppressive therapy had a decreased risk of developing PD (106). Interestingly, the greatest risk reduction was conveyed by drugs affecting T cells such as the inosine monophosphate dehydrogenase inhibitors, azathioprine and mycophenolat mofetil (106), while a protective effect was revealed for corticosteroids. However, this finding might be biased by smoking behavior. Smoking is described as a possible protective factor for PD, and smokers frequently need corticosteroids due to pulmonary maladies (106, 107). Moreover, cigarette smoke induces chronic inflammation, which results in immune cell exhaustion and generally attenuates the function of many immune responses (108). Therefore, smoking can be considered an immune-inhibitory activity (109, 110). The potential effectiveness of anti-inflammatory treatment is further exemplified by a lowered risk for developing PD in patients receiving immunosuppressive therapy (111).

Accordingly, patients with inflammatory bowel disease (IBD) receiving an anti-TNF treatment had a 78% reduction in the incidence of PD compared to IBD patients who did not receive this therapy (112). However, the lack of direct comparison between the incidence of PD in treated IBD patients compared with non-IBD patients (112) means that the ability of anti-TNF treatment to reduce PD incidence in non-IBD patients remains unanswered. Other research has indicated that severity of IBD is inversely associated with the risk of developing PD (-15%) (113). However, although patients suffering from severe IBD are more likely to receive anti-inflammatory drugs, immune-modulatory treatment was not directly assessed. Accordingly, further studies are necessary to verify a positive or negative correlation between PD and anti-inflammatory treatment, both for IBD and for non-IBD patients.

One of the most frequently used anti-inflammatory drugs is ibuprofen, a reversible cyclooxygenase-2 (COX-2) inhibitor and non-steroidal anti-inflammatory drug (NSAID) (114). Intriguingly, ibuprofen users showed a lower risk for PD, whereas no protective effect could be demonstrated for acetylsalicylic acid (ASA) (114). In a prospective study of subjects without PD who were assessed at baseline for NSAID intake, 0.2% developed PD during a 6-year follow-up (115). A significant dose-response relationship between ibuprofen intake (tablets/week) and reduced PD risk was found. Again, this effect was not observed for ASA, other NSAIDs (e.g., indomethacin), or acetaminophen. Although all NSAIDs and ASA are known to inhibit COX-2, only ibuprofen was shown to have some impact on PD prevention. Pre-clinical findings from *in vitro* models indicate that ibuprofen prevents oxidative damage induced by LPS injection and better protects dopaminergic neurons against glutamate neurotoxicity than ASA (116, 117). The neuroprotective effects of ibuprofen have been confirmed in studies that considered confounder variables of PD, such as age, smoking, and caffeine intake (115, 118). However, the putative neuroprotective effect of ibuprofen in PD remains controversial; other research focusing on all NSAIDs rather than on ibuprofen alone found that NSAIDs had no neuroprotective effects (107). In this context, it is noteworthy that ibuprofen is known to reduce inflammation *via* inhibition of COX-2, but also *via* modulation of the angiotensin pathway by shifting the ACE/ACE-2 (angiotensin converting enzyme) receptor ratio towards ACE-2 (119, 120) (discussed in detail in Section 4.2).

Drugs such as β-agonists and β-antagonists have been shown to affect the incidence of PD in opposing directions, with β-agonists seeming to be somewhat protective (121, 122). However, further evidence is needed to confirm whether β-agonists might act directly on immune cells and are thus capable of modulating PD-related inflammation (122). It should be noted that β-agonists are often used in patients suffering from chronic obstructive pulmonary disease, a common consequence of frequent smoking. The fact that smoking was reported to lower the incidence of PD in one study represents an epidemiological bias (121).

Given that observational studies have yielded controversial data regarding the neuroprotective effects of different drugs and immunomodulators (123–128), randomized, controlled trials are needed to better decipher which drugs have distinct neuroprotective effects in PD.

### 2.5 Current Clinical Trials for Anti-Inflammatory Disease Modification in PD

Current clinical trials on anti-inflammatory therapy in PD focus on the treatment of glucose metabolism, improvement of oxidative stress, and regulation of gut microbiota.

#### 2.5.1 Regulation of Glucose Metabolism

Impaired insulin signaling resulting in altered energy balance and impaired cell repair mechanisms has been observed in PD patients (129–131). Glucagon-like peptide-1 (GLP-1) is a growth factor and insulin-stimulating hormone that can activate the same effector molecules as insulin, and its receptor (GLP1R) is expressed in neurons. In addition to the known effects of GLP1R on blood sugar regulation and stimulation of the hypothalamus to regulate appetite, studies have shown that GLP1R agonists have neuroprotective effects in different animal models of PD. One agonist, exendin-4, can enhance cognitive function (132) and attenuate neurodegeneration in 6-hydroxydopamin (6-OHDA) rats (133). In 1-methyl-4-phenyl-1,2,3,6-tetrahydropyridine (MPTP)-treated mice, the newer GLP-1 mimetics liraglutide and lixisenatide can improve motor impairment and reduce dopaminergic cell loss in the SN (134, 135). In a human A53T αSYN (hA53T-αSYN) transgenic mouse model, a long-acting GLP1R agonist reduced behavioral deficits and neurodegeneration (136). It is noteworthy that activation of GLP1R can inhibit the production of proinflammatory cytokines in microglia, such as TNF and IL-1B (137, 138). The neuroprotective effect of semaglutide treatment is currently under investigation (139).

#### 2.5.2 Regulation of Gut Microbiota

The gastrointestinal (GI) tract and CNS have complex mutual interactions (140) that are affected by the intestinal flora (141–143). Therefore, a healthy and stable intestinal flora is important for immunity, homeostatic balance of barrier integrity, and metabolism (144). Interestingly, changes in the intestinal flora may alter nerve development and even cause neurodegenerative disorders (145). Conversely, traumatic brain injury may lead to impaired intestinal barrier function (146, 147). GI dysfunction in PD patients, particularly obstipation, is commonly known to be one of the initial symptoms that precedes motor impairment (148). Changes in intestinal microbial flora can alter the barrier function and permeability of the gut and subsequently influence the immune system (140, 149), and have been linked to PD (149). It is noteworthy that pαSYN can be found in the enteric nervous system (150).

A speculative hypothesis is that the methylation status of the SNCA gene might be influenced by the gut microbiome. It is accepted that duplications and triplications of the SNCA gene confer a higher risk of developing PD (151, 152). Decreased methylation of the SNCA gene, which would lead to increased transcription, could likewise affect αSYN expression and influence the risk of PD (153). Postulating a potential role of the gut microbiota as an epigenetic factor for DNA methylation, an ongoing clinical study is investigating the composition of the GI bacteria in the stool of PD patients and healthy controls by extracting total bacterial DNA from the samples (154).

#### 2.5.3 Regulation of Oxidative Stress

Oxidative stress in the brain plays a key role in the development of PD (155, 156), and results from an imbalance between reactive oxygen species (ROS) and the ability to scavenge reactive intermediates (157). ROS, which include superoxide, nitric oxide, hydroxyl radical, hydrogen peroxide, and peroxynitrite, can be generated from various sources including the electron transport chain or activated immune cells. Interestingly, increased oxidative stress seems to be target-specific and, in the case of PD, related to LP and the pαSYN contained in Lewy bodies, where pαSYN nitration (an oxidation marker) was found to be elevated (158–160). Furthermore, the SN of early PD patients has been found to contain reduced amounts of the antioxidant glutathione (GSH), which correlated with PD severity (159).

Increasing the availability of reduced GSH N-acetylcysteine (NAC) can potentially reduce the damage to neurons caused by oxidative stress. In a current clinical trial, NAC is being used to protect PD patients from oxidative damage (161). Aside from NAC, other supplements with antioxidative properties are under investigation. In the CNS, vitamin B_3_ (niacin) is considered a key mediator of neuronal development and survival (162). Niacin is a cofactor of nicotinamide adenine dinucleotide and NADPH, which are necessary for the scavenging of oxidants and needed for GSH regeneration (157). Niacin can improve the integrity of mitochondria and hence the energy supply of neurons. By enhancing antioxidant defense mechanisms, niacin can protect the normal structure and function of neurons exposed to oxidative stress, thereby delaying the progression of PD (163). Furthermore, niacin binds to the G-protein-coupled hydroxycarboxylic acid receptor 2, which is upregulated in a proinflammatory environment and which may downregulate inflammation (164–166). Since PD patients are known to have low levels of niacin due to mitochondrial malfunctioning, supplementation of niacin could be beneficial (166). A clinical trial is evaluating the effectiveness of niacin or niacinamide supplementation on inflammation and severity of PD symptoms (167).

## 3 Animal Models and Their Contribution Towards Understanding PD Pathomechanisms

Animal models play an important role in understanding PD pathophysiology, including neuroinflammatory processes. Next to the 6-OHDA model (168), one of the most often used PD models is induced by MPTP delivery (169), which persists extensively in the SN and results in PD symptoms with inhalation and cutaneous contact (169). Immune responses in the various MPTP models used are, however, quite different.

### 3.1 Innate Immune Responses in Animal Models for PD

**Figure 1:**
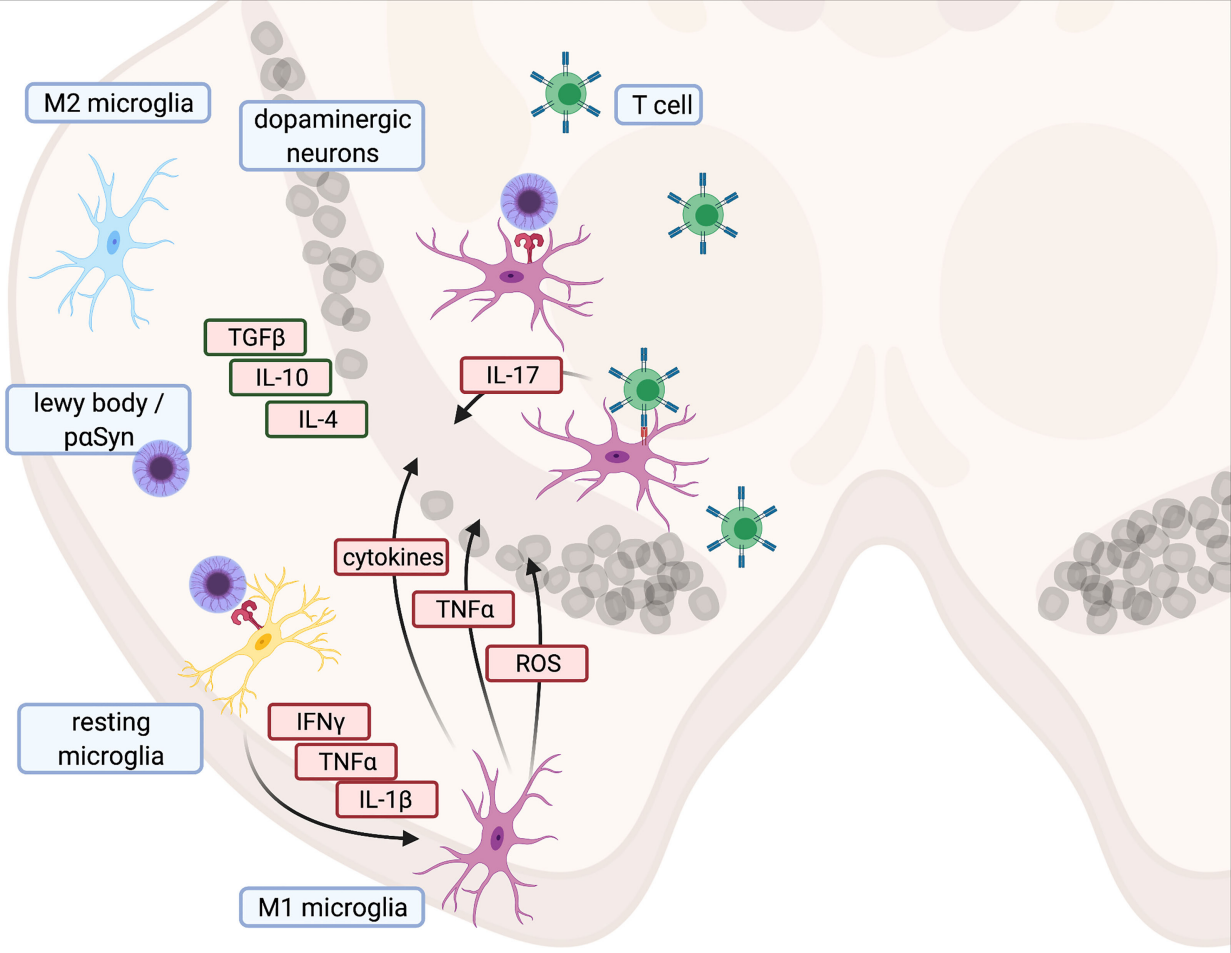
Illustration of proinflammatory cascade and main cytokines contributing to cell death in Parkinson’s disease - Created with BioRender.com.

Toxins such as MPTP, rotenone, and 6-OHDA act as DAMPs and initiate a strong innate immune response with microglia activation and subsequent neurodegeneration in the SN (170–172). This immune response resembles the microglia activation found in human brain autopsies (21, 173). In the 6-OHDA model, neurodegeneration is accompanied by a gradual repolarization of microglia from an anti-inflammatory M2 to a pro-inflammatory M1 phenotype (174). Cytokine production by M1 cells is intracellularly initiated by NFKB (53, 175, 176), which in turn induces interleukin and procaspase-1 transcription. Together with the inflammasome NLRP3, caspase-1 activates IL-1B. Moreover, other proinflammatory proteins such as iNOS and TNF are also released from M1 cells (53, 175, 176) and contribute to neurodegeneration in PD (177). Consequently, inhibition of NFKB protects MPTP mice from neurodegeneration (178). M2 macrophages, in contrast, contribute to neuroprotection and release neurotrophic factors (176) (**Figure 1**). Since TLR-4 knock-out reduced the number of MHC-II^+^ microglial cells and partially protected MPTP-treated mice from dopaminergic degeneration in the SN, MPTP triggers also neuroinflammation *via* TLR-4 (179). However, the canonical ligand for TLR-4 is LPS, which is commonly found on cell walls of gram-negative bacteria (180) In LPS-driven models, LPS is either systemically administered or focally injected into the SN (181). Since microglia activation after delivery of LPS or another of the aforementioned toxins results in the same downward cascades, all of these models lend themselves to studying innate immune responses (181–183). However, in mice lacking the mitophagy inducers PINK and Parkin, LPS triggers the release of mitochondria-derived vesicles (184, 185). This can prime CD8^+^ T cell responses against mitochondrial antigens (184, 185). External stressors such as LPS can thus also induce adaptive immune responses, in particular in transgenic models with impaired mitochondrial functions.

For MPTP, but not 6-OHDA, a Lewy body-like pathology has been found in non-human primates (186). An even more prominent Lewy body-like histopathology was observed in viral vector-based models overexpressing human mutations of αSYN (e.g., A53T) (187). Furthermore, viral vector-based models demonstrate a similar innate immune response, as seen in toxin-based models. pαSYN, which is believed to enter the cell *via* TLR-2 to unleash its toxic potential (53, 188, 189), thus apparently acts like a DAMP (190). Interestingly, orthotopic injection of an αSYN-encoding AAV into the SN only induced neurodegeneration in the presence of Fc gamma receptors (191), which implies an antibody-dependent activation of the complement cascade and/or antibody-dependent cellular cytotoxicity that may be mediated by microglia, for example (192). However, unlike astrocytes, microglia do not directly contribute to a proinflammatory environment after exposure to pαSYN (193). Nevertheless, progressive neurodegeneration can be induced by microglia-specific overexpression of pαSYN in the absence of pαSYN in dopaminergic cells (194). The critical role of CD4^+^ T cells was confirmed by showing that virus-based αSYN-overexpression can only induce microglia activation and neurodegeneration when MHC-II is present (195). Therefore, viral vector-based models require a complex interplay between different types of immune cells, which may also best reflect observations made in human PD.

Since animal research in PD has focused mostly on alterations within the CNS, our understanding of peripheral innate and adaptive immune processes in PD pathology is still limited. Recent studies have provided evidence that increased gut permeability and gut microbiota are likely to contribute to PD development (149, 196). This has resulted in PD becoming more and more accepted as a systemic disease (197). For instance, overexpression of human TNF (hTNF) in a mouse model leads to microglia activation in various regions of the brain (198, 199), while immunomodulation with infliximab could attenuate microglia activation (199). Since infliximab cannot penetrate the blood-brain barrier, this finding indicates that effective treatment of neuroinflammation could be achieved by modulating peripheral immune mechanisms.

### 3.2 Adaptive Immune System in Animal Models for PD

Several animal models support the hypothesis that the adaptive immune response in PD is mainly T cell driven, whereas the role of B cells remains enigmatic (41, 42, 73, 75, 88, 200). Nevertheless, the mechanisms of T cells vary across different animal models (42, 201).

#### 3.2.1 MPTP Model

Variations in frequency and dosage of MPTP injections in mice result in different PD models with diverse disease kinetics. The subacute MPTP model is generated by administering MPTP once daily over five consecutive days, whereas an acute model is induced by delivering MPTP every two hours for four times in only one day (202). For the acute model, it is important to note that in contrast to humans, the initial immune response decreases significantly within 30 days, and MPTP-injected mice may recover over time (203). Moreover, pαSYN is not found in this model *per se*. However, if the adaptive immune response in the acute model is maintained by peripherally administering cytokines such as RANTES (Regulated on Activation, Normal T cell Expressed and Secreted) and eotaxin twice a week, the inflammation is sustained and pαSYN accumulates in the SN of the rodents (203). In this model, pαSYN formation is linked to a sustained proinflammatory environment.

CD4^+^ and CD8^+^ T cell infiltration in the SN of acute MPTP mice supports the idea that T cells contribute to neurodegeneration in this PD model (42). CD4^+^ or recombinant activating gene 1 (RAG1)-deficient mice, which lack mature lymphocytes, show attenuated neurodegeneration upon treatment with MPTP. In contrast, neurodegeneration is unrestrained in CD8^+^ T cell-deficient mice (42). Blockade of RANTES and eotaxin in the MPTP mouse and non-human primate model reduces microglia activation, CD4^+^ and CD8^+^ T cell infiltration, and neurodegeneration (204, 205). Continuous T cell trafficking and induction of pαSYN in the SN of MPTP mice is also driven by supplementation of RANTES and/or eotaxin, but not by TNF and IL-1B (203). Furthermore, MPTP-treated mice show altered and dysfunctional CD4^+^CD25^+^ T_reg_ cells. Administration of natural T_reg_ cells leads to attenuation of SN degeneration (206). As Th17 cells appear to contribute to nigrostriatal degeneration (206, 207), the beneficial effect of T_reg_ may be due to their ability to inhibit TH17 cells. However, while mechanistic insights from the aforementioned studies are based on the acute MPTP model, CD4^+^ T cell infiltration occurs earlier in the subacute model (208). Given the differences between the models, the choice of the most appropriate model may determine the outcome (209).

#### 3.2.2 6-OHDA Model

This model is generated by injecting 6-OHDA either directly into the SN, the medial forebrain bundle, or the striatum (168, 210, 211). The initial cytotoxic effect of 6-OHDA is known to be mediated *via* oxidative stress (212) and impaired mitochondrial function (213). Data on the role of the adaptive immune response in the 6-OHDA model are still scarce. But it is known that 6-OHDA administration leads to infiltration of CD4^+^ and T_reg_ cells in the CNS (76, 174). In contrast to MPTP, 6-OHDA must only be administered once to induce continuous neuroinflammation and neurodegeneration (174, 201). Apart from neuroinflammation, 6-OHDA-treated rats also show decreased circulating T_reg_ cells, indicating that this model can reflect aspects of human PD (174). However, while CD4^-/-^ or Rag1^-/-^ knock-out mice injected with MPTP were partly protected from severe neurodegeneration (42), 6-OHDA-treated Rag1^-/-^ mice showed even more pronounced neurodegeneration in the SN than immune-competent mice (201) or rats (214). Accordingly, the adaptive immune system is mostly neuroprotective in the 6-OHDA rodent model. Thus, toxin-based models provide contradictory data on the role of the adaptive immune system. Based on the epidemiological data linking PD and adaptive immune responses in humans, these models do not seem to adequately recapitulate the pathomechanisms underlying the human disease.

#### 3.2.3 Viral Vector and Preformed Fibrils Models

Creating a model that shows a large overlap with the human phenotype and pathophysiology is the main objective when generating new animal models. Regarding chronic disease progression, mild-to-moderate nigrostriatal degeneration, Lewy-like pathology, and neuroinflammation, the AAV1/2-A53T-αSYN model resembles human PD much more closely than conventional toxin-induced models (75, 187, 215). Recently, CD4^+^ and CD8^+^ cells infiltrating the SN were found in the AAV1/2-A53T-αSYN model (75). Ten weeks after AAV1/2-A53T-αSYN injection, CD4^+^ and CD8^+^ cells in the spleen were also activated. Moreover, RAG deficiency protected dopaminergic SN neurons, indicating that CD4^+^ and CD8^+^ crucially contribute to neurodegeneration in this model (75). This reflects findings made in PD patients, thereby demonstrating a higher immune-related face validity of the AAV1/2-A53T-αSYN mouse model compared to the toxin-induced MPTP and 6-OHDA models. Comparable results, including gradual dopaminergic cell loss and CD3^+^ infiltration in the SN, were also achieved by viral vector-based application of human αSYN in the SN of mice (216, 217) or by directly injecting preformed αSYN fibrils (αSYN-PFF) into the striatum of mice (62). The immune response triggered by the presence of pαSYN is thus conserved and comparable between different models. This is in line with the recently revealed role of αSYN as a critical mediator of inflammatory and immune responses (18).

Aside from PD models with direct injection of pαSYN or αSYN-PFF into the brain, there are also models based on peripheral injection of αSYN-PFF. Intriguingly, spread of pαSYN from the periphery to the brain is observed, accompanied by motor deficits (218, 219). However, in a more recent study where strong involvement of the adaptive immune response in the brain was confirmed, further inflammatory stress by LPS was required to achieve spreading of pαSYN into the brain (220). Accordingly, ongoing neuroinflammation contributes to the progression and maintenance of PD (197, 221–225).

#### 3.2.4 Conclusion for the Role of the Immune System in PD Animal Models

These animal models provide substantial support for the hypothesis that the immune system participates in the neurodegeneration observed in PD. However, for investigating adaptive immunity, choosing the right animal model is key. Central and peripheral immune responses must both be considered. Finally, since the overall role of the immune response in PD animal models is still understudied, interactions between pαSYN, microglia, astrocytes, the adaptive immune system, and peripheral inflammation need to be explored.

## 4 Signaling Cascades Related to PD and Neuroinflammation as Possible Therapeutic Targets

Pathophysiology of PD is manifold and includes several well-investigated pathways, while others may still be unknown. There are several important signaling pathways related to inflammation and neurodegeneration.

### 4.1 COX-2 and Disease Modification With NSAIDs

ASA is a standard drug for secondary prophylaxis of ischemic stroke and a potent non-competitive inhibitor of COX-2. Since COX-2 is expressed in many cell types, including platelets and microglia, it is involved in many physiological processes. Intriguingly, the protective role of COX-2 inhibitors on neuroprotection remains controversial. An early administration of high ASA doses showed a neuroprotective effect in MPTP mice, while other COX-2 inhibitors including ibuprofen did not (226). In contrast, the protective effect of ASA in PD patients is comparatively low, while ibuprofen is associated with decreased disease risk compared to other NSAIDs (114, 115). Furthermore, some studies have shown that NSAID intake has no effect on PD development, but instead correlates with an increased risk for PD (107, 227). It is important to note that none of these studies compared equivalent dosages. In MPTP mice, however, COX-2 is upregulated and known to be involved in the oxidation of dopamine, forming dopamine quinone (DAQ) (202). Interestingly, COX-2-deficient mice are protected against induction of PD, which was not explained by reduced inflammation but by prevention of DAQ formation (202, 228, 229). DAQ conjugates with GSH to form GSHDAQ. After conversion to 5cysDAQ, GSHDAQ inhibits mitochondrial complex I. In turn, this leads to decreased degradation of ROS and increased DAQ levels. Subsequently, this vicious circle results in microglia and astrocyte activation and a shift towards a proinflammatory state (175), accompanied by exhaustion of the pentose-phosphate pathway (PPP). However, there are multiple pathways that result in upregulation of COX-2 in brain neurons, including increased N-methyl-D-Aspartate (NMDA) receptor-dependent activity (230), cerebral ischemia, seizures, and diseases such as AD or PD (231). Another important function of COX-1 and COX-2 is the further processing of arachidonic acid. COX-1 induction leads to a substantial increase in thromboxane A2 (TXA_2_) in human endothelial cells, while induction of COX-2 by IL-1B leads to a minor increase in TXA_2_ but a substantial induction of the proinflammatory proteins prostacyclin (PGI_2_) and prostaglandin E2 (PGE_2_) (232).

PGI_2_ and PGE_2_ are physiologically involved in body temperature regulation. PGI_2_ and TXA_2_ have important roles in the maintenance of vascular homeostasis and can mainly be described as agonist and antagonist (233). Among the different PGE_2_ receptors (EP1-4), EP2 contributes to neurodegeneration in the presence of pαSYN in PD (234). EP2-deficient microglia from mice showed increased clearance of pαSYN on brain slices from Lewy-body patients. Furthermore, EP2^-/-^ MPTP mice were spared from neurodegeneration in the SN (234). However, this mechanism only seems to apply for neurotoxicity associated with chronic inflammatory processes. In acute pathological processes such as brain ischemia or NMDA-induced toxicity, EP2 activation exerts protein kinase A-dependent protective effects (231). Interestingly, EP2 is also neuroprotective in the 6-OHDA primary cell culture (235). The cause of this dichotomous action of EP2 is not yet understood (231).

Beside possible neuroprotective effects, COX-2 inhibitors increase intestinal permeability that correlates with their potency to inhibit COX-2, thus ultimately leading to local inflammatory side effects (236, 237). Furthermore, since increased intestinal permeability is discussed as an important pathological axis of PD development, prolonged treatment of PD with potent NSAIDs is not advisable, which could explain the controversial results (149, 196).

### 4.2 Oxidative Stress, PPP, and Renin-Angiotensin System

#### 4.2.1 Oxidative Stress and PPP

Under steady-state conditions, ROS are maintained in a stable range and participate in the regulation of cell growth, differentiation, apoptosis signals, and enzyme activity by modulating the production of ceramide, kinase regulatory proteins, and activation of transcription factor NFKB (238, 239). Oxidative stress is a potent and highly conserved defense mechanism against intruders such as bacteria or fungi (240). By stimulating the production of inflammatory factors, ROS play an essential role in pathogen clearance (238). Free radical scavengers such as GSH act as a counterpart to ROS and protect cells from excessive oxidative stress. Maintaining the reduced state of GSH by oxidation of NADPH is therefore important for tissue homeostasis. Under pathological conditions, such as in PD, the level of ROS outpaces the compensatory mechanisms. ROS are located upstream of many inflammatory signaling pathways, upregulate the expression of other inflammatory factors, interfere with signaling pathways that regulate growth factors, and often alter signal transduction. High levels of ROS can be generated (241–244) by M1 microglia, but also the renin-angiotensin system (RAS) or NADPH-oxidase and its homologues, the so-called NOX enzymes (240). High ROS concentrations may cause apoptosis, structural damage, and cell death (159). ROS can therefore directly exert neurotoxicity and amplify neurotoxic inflammatory responses (157, 159). Understanding the role of pathways such as the PPP, which expurgate excessive amounts of ROS, might be crucial for finding an effective treatment against PD. The PPP connects glucose metabolism with NADPH production and nucleotide precursor synthesis. In this pathway, glucose-6-phosphate (G6P) is metabolized to produce NADPH and ribose-5-phosphate. In nearly all cells, the main task of PPP is to provide ribose-5-phosphate. In neurons, however, its main task is to provide NADPH to eliminate ROS (245). The definite role of PPP in PD remains still uncertain (245), even though there is increasing evidence that PD is associated with disorders of glucose metabolism (246–248).

The rate-limiting enzyme of the oxidative part of the PPP is G6P dehydrogenase (G6PD), which catalyzes the reaction between G6P and NADP^+^. Through consumption of NADPH, oxidative stress leads to the accumulation of large amounts of NADP^+^ and subsequently to the activation of G6DP due to the increased concentration of oxidants (249). In MPTP mice, neurodegeneration was shown to correlate with NADPH oxidase upregulation (242). Moreover, G6PD expression was positively associated with microglial activation and dopaminergic neurodegeneration (245), indicating that oxidative stress is closely linked to neurodegeneration.

Another important protein related to oxidative stress is GSH, an important antioxidant in brain neurons. The level of GSH in cultured cells is directly related to the production of NADPH by PPP (250). Increased ROS and decreased GSH levels have been described in many PD models and patients (155, 251–254).

Maintaining or adding NADPH in therapeutic dosages might help to overcome high levels of ROS, keep a stable level of GSH in the brain, and finally result in neuroprotection.

#### 4.2.2 Renin-Angiotensin(-Aldosterone) System

**Figure 2:**
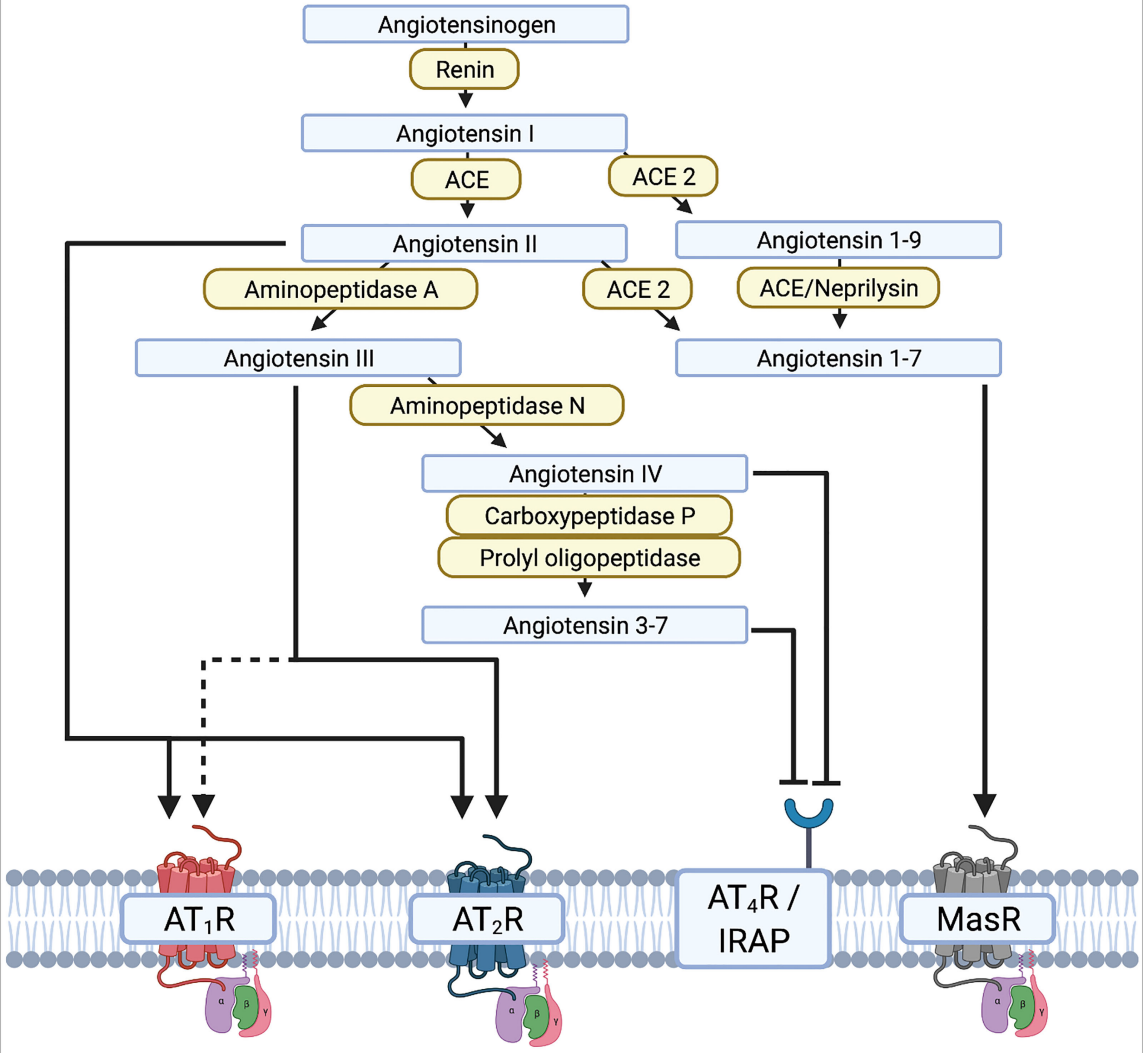
Illustration of the renin-angiotensin system (RAS) and the different interactions of the renin-angiotensin-[aldosterone] system RAAS substrates on receptors. - Created with BioRender.com.

The renin-angiotensin[-aldosterone] system (RA[A]S) is well-known in the context of blood pressure regulation (255). Stimuli such as increased activity of kidney baroreceptors, low plasma sodium levels, and activation of β1-receptors result in release of renin from the kidney into the blood circulation. Renin then cleaves liver-synthesized angiotensinogen into angiotensin I (AngI). Subsequently, AngI is cleaved *via* ACE to angiotensin II (AngII). Binding of AngII to the G-protein-coupled AngII receptor type 1 (AT_1_R) is essential for blood circulation. AT_1_Rs are commonly expressed in the vascular system, the heart, and the brain. Aside from binding to AT_1_R, AngII also induces release of aldosterone from the adrenal cortex (256). Drugs for the treatment of arterial hypertension therefore interfere with the RA[A]S by inhibiting renin, ACE, AT_1_R, or the aldosterone receptor (257). Investigations into the role of the RA[A]S in the brain, and more specifically in dopaminergic neurons, have deepened our understanding of neurodegeneration in PD. Different groups have demonstrated in both 6-OHDA and MPTP mice that neurodegeneration can be accelerated or decelerated through AT_1_R activation or blockade, respectively (243, 255, 258, 259). However, the RAS includes many complex crosslinks with pro- and anti-inflammatory endpoints (**Figure 2**). The main axis consists of core substrates including angiotensinogen, AngI/II, and renin. AngII mainly binds AT_1_R, but also the AngII receptor (AT_2_R), which initiates anti-inflammatory and anti-oxidative cascades (123, 260). This contrasts with AT_1_R, which initiates and maintains neuroinflammation and oxidative stress (260–262). AngII can be cleaved to AngIII by aminopeptidase-A, and further to AngIV and Ang3-7 by aminopeptidase N or carboxypeptidase P and prolyl-oligopeptidase, respectively. AngIII binds to AT_1_R and AT_2_R, whereas AngIV has a low affinity to AT_1_R and AT_2_R but a high affinity to the AngIV receptor (**Figure 2**). AngIV and analogues were shown to have a positive effect on synaptogenesis and improved clinical outcomes after stroke and subarachnoid hemorrhage by increasing cerebral blood flow (263–266).

The neurodegenerative effect mediated by AngII and the AngII-AT_1_R complex are mediated by an increased release of ROS *via* the NADPH-oxidase complex in microglia and dopaminergic neurons, resulting in a vicious proinflammatory circle (123). The increased release of ROS is mediated by increased nuclear expression of AT_1_R and NOX in dopaminergic neurons of PD patients, resulting in oxidative DNA damage and cell loss (267). In contrast, the cytoplasmic and membrane expression of AT_1_R is reduced (267). Under normal conditions, the AT_1_R-NOX interaction maintains cell homeostasis and physiological levels of dopamine. The pathological shift of AT_1_R expression from the membrane/cytoplasm to the nucleus reflects the disease process and contributes to secondary neurodegeneration due to increased oxidative stress. Interestingly, this shift can be observed relatively early. An altered ratio of membrane/cytoplasm to nuclear AT_1_R expression has already been observed in patients with a neuropathological diagnosis of PD who had not yet developed clinical manifestations or significant dopaminergic neurodegeneration (267). Yet to date, a potential re-distribution of other AT receptors in the brain of human PD patients has rarely been explored. However, ACE2 cleaves AngII and AngI to Ang1-9 and Ang1-7, respectively, and thereby shifts the RAS away from the proinflammatory main axis to the more anti-inflammatory side axis. Altogether, the RAS is a complex system that may be amenable to therapeutic modulation. The potential of targeting the RAS in PD and AD has therefore been discussed in considerable detail in dedicated review articles (123, 261).

Of note, unwanted alterations in the subtle balance between the pro- and the anti-inflammatory axis of the RAS occur during COVID-19 infections, where ACE2 is the main entry receptor for the pathogenic SARS-CoV-2 virus (268). Neurological consequences of the strong inflammation observed in severe COVID-19 remain to be studied.

## 5 Conclusions/Outlook

Even 200 years after the first description of PD, its pathophysiology is still far from being fully understood. Fortunately, research towards a causal treatment of PD has made substantial progress in the last 15 years. Realizing that (systemic) inflammation plays an essential role of PD, at least in its maintenance, might pave the way for new therapeutic pathways. Knowledge transfer from classical inflammatory diseases or diseases that lead to inflammatory processes will likely have a major impact on the therapy of PD, and allow new development and/or a repurposing of drugs that are already available. In addition, research in early diagnostics for PD is important. Ideally, neurodegeneration can be halted by initiating treatment before severe symptoms have developed. Skin biopsy or sleep analysis, along with new serum/liquor parameters, might lead to a sensitive and specific early diagnosis of PD. In turn, this should open up an earlier treatment window for future PD patients. Treating early with well-tolerated neuroprotective/anti-inflammatory agents certainly holds great promise for this devastating disease.

## Author Contributions

AG - Conceptualization, Visualization, Writing - original draft, Writing – review & editing. RM - Writing - review & editing. JWu - Writing - original draft. JWi - Writing - review & editing. CWI - Conceptualization, Supervision, Writing - review & editing. All authors contributed to the article and approved the submitted version.

## Funding

CWI is supported by the Interdisciplinary Center for Clinical Research (IZKF) at the University of Würzburg (A-303 to CWI, A-421 to CWI and JWi, N-362 to CWI), by the Deutsche Stiftung Neurologie and the ParkinsonFonds Germany and by the University Research Funds of the State of Bavaria. Moreover, CWI is supported by the Deutsche Forschungsgemeinschaft (DFG, German Research Foundation) Project-ID 424778381-TRR 295 (A06) and the VERUM Foundation. CWI and JWi are supported by Aeterna Zentaris. AG is supported by Interdisciplinary Center for Clinical Research (IZKF) at the University of Würzburg (Z-2/79) and is a Gerok-Position holder funded by the CRC TRR 295 ‘Retune’. RM is funded by the Alexander von Humboldt-Stiftung.

## Conflict of Interest

The authors declare that the research was conducted in the absence of any commercial or financial relationships that could be construed as a potential conflict of interest.

## Publisher’s Note

All claims expressed in this article are solely those of the authors and do not necessarily represent those of their affiliated organizations, or those of the publisher, the editors and the reviewers. Any product that may be evaluated in this article, or claim that may be made by its manufacturer, is not guaranteed or endorsed by the publisher.

## References

[1] Parkinson J. An Essay on the Shaking Palsy. Needly and Jones, London: Whittingham and Rowland for Sherwood (1817).

[2] Goedert M Compston A. Parkinson’s Disease — the Story of an Eponym. Nat Rev Neurol (2017) 14(1):57–62. doi: 10.1038/nrneurol.2017.165 29217826

[3] Goetz CG. The History of Parkinson’s Disease: Early Clinical Descriptions and Neurological Therapies. Csh Perspect Med (2011) 1(1):a008862. doi: 10.1101/cshperspect.a008862 PMC3234454 22229124

[4] Walusinski O. Jean-Martin Charcot and Parkinson’s Disease: Teaching and Teaching Materials. Rev Neurol (2018) 174(7–8):491–505. doi: 10.1016/j.neurol.2017.08.005 29653830

[5] Hoehn MM Yahr MD. Parkinsonism: Onset, Progression, and Mortality. Neurology (1967) 17(5):427–7. doi: 10.1212/WNL.17.5.427 6067254

[6] Armstrong MJ Okun MS. Diagnosis and Treatment of Parkinson Disease. JAMA (2020) 323(6):548. doi: 10.1001/jama.2019.22360 32044947

[7] Berg D Postuma RB Adler CH Bloem BR Chan P Dubois B. MDS Research Criteria for Prodromal Parkinson’s Disease: MDS Criteria for Prodromal Pd. Movement Disord (2015) 30(12):1600–11. doi: 10.1002/mds.26431 26474317

[8] Braak H Tredici KD Rüb U de Vos RAI Steur ENHJ Braak E. Staging of Brain Pathology Related to Sporadic Parkinson’s Disease. Neurobiol Aging (2003) 24(2):197–211. doi: 10.1016/S0197-4580(02)00065-9 12498954

[9] Braak H Braak E. Pathoanatomy of Parkinson’s Disease. J Neurol (2000) 247(Suppl 2):II3–10. doi: 10.1007/PL00007758 10991663

[10] Surmeier DJ Obeso JA Halliday GM. Selective Neuronal Vulnerability in Parkinson Disease. Nat Rev Neurosci (2017) 18(2):101–13. doi: 10.1038/nrn.2016.178 PMC5564322 28104909

[11] Braak H Ghebremedhin E Rüb U Bratzke H Tredici KD. Stages in the Development of Parkinson’s Disease-Related Pathology. Cell Tissue Res (2004) 318(1):121–34. doi: 10.1007/s00441-004-0956-9 15338272

[12] Dijkstra AA Voorn P Berendse HW Groenewegen HJ Bank NB Rozemuller AJM. Stage-Dependent Nigral Neuronal Loss in Incidental Lewy Body and Parkinson’s Disease. Movement Disord (2014) 29(10):1244–51. doi: 10.1002/mds.25952 24996051

[13] Cheng J Lu Q Song L Ho MS. α -Synuclein Trafficking in Parkinson’s Disease: Insights From Fly and Mouse Models. Asn Neuro (2018) 10:175909141881258. doi: 10.1177/1759091418812587 PMC6259071 30482039

[14] Kalia LV Lang AE. Parkinson’s Disease. Lancet (2015) 386(9996):896–912. doi: 10.1016/S0140-6736(14)61393-3 25904081

[15] Killinger BA Kordower JH. Spreading of Alpha-Synuclein - Relevant or Epiphenomenon? J Neurochem (2019) 150(5):605–11. doi: 10.1111/jnc.14779 31152606

[16] Visanji NP Brooks PL Hazrati L-N Lang AE. The Prion Hypothesis in Parkinson’s Disease: Braak to the Future. Acta Neuropathol Commun (2013) 1(1):2. doi: 10.1186/2051-5960-1-2 24252164 PMC3776210

[17] Hijaz BA Volpicelli-Daley LA. Initiation and Propagation of α-Synuclein Aggregation in the Nervous System. Mol Neurodegener (2020) 15(1):18. doi: 10.1186/s13024-020-00368-6 PMC7060612 32143659

[18] Alam MM Yang D Li X-Q Liu J Back TC Trivett A. Alpha Synuclein, the Culprit in Parkinson Disease, Is Required for Normal Immune Function. Cell Rep (2022) 38(2):110090. doi: 10.1016/j.celrep.2021.110090 35021075 PMC10258816

[19] Ransohoff RM. How Neuroinflammation Contributes to Neurodegeneration. Science (2016) 353(6301):777–83. doi: 10.1126/science.aag2590 27540165

[20] Rogers J Webster S Lue L-F Brachova L Civin WH Emmerling M. Inflammation and Alzheimer’s Disease Pathogenesis. Neurobiol Aging (1996) 17(5):681–6. doi: 10.1016/0197-4580(96)00115-7 8892340

[21] McGeer PL Itagaki S Boyes BE McGeer EG. Reactive Microglia are Positive for HLA-DR in the Substantia Nigra of Parkinson’s and Alzheimer’s Disease Brains. Neurology (1988) 38(8):1285–5. doi: 10.1212/WNL.38.8.1285 3399080

[22] McGeer PL McGeer EG. Targeting Microglia for the Treatment of Alzheimer’s Disease. Expert Opin Ther Tar (2014) 19(4):497–506. doi: 10.1517/14728222.2014.988707 25435348

[23] Wang S Breskovska I Gandhy S Punga AR Guptill JT Kaminski HJ. Advances in Autoimmune Myasthenia Gravis Management. Expert Rev Neurother (2018) 18(7):573–88. doi: 10.1080/14737175.2018.1491310 PMC6289049 29932785

[24] Venkataramaiah S Kamath S. Management of Myasthenia Gravis. J Neuroanaesth Crit Care (2019) 06(02):153–9. doi: 10.1055/s-0039-1689739

[25] Hardy TA Reddel SW Barnett MH Palace J Lucchinetti CF Weinshenker BG. Atypical Inflammatory Demyelinating Syndromes of the CNS. Lancet Neurol (2016) 15(9):967–81. doi: 10.1016/S1474-4422(16)30043-6 27478954

[26] Hartung H-P Aktas O. Old and New Breakthroughs in Neuromyelitis Optica. Lancet Neurol (2020) 19(4):280–1. doi: 10.1016/S1474-4422(20)30062-4 32199085

[27] Wesley S Hafler DA. Chapter 51 - Multiple Sclerosis In: Rose NR Mackay IR. The Autoimmune Diseases (Sixth Edition). Academic Press. (2020). p. 961–986. doi: 10.1016/B978-0-12-812102-3.00051-8

[28] Chamberlain JL Huda S Whittam DH Matiello M Morgan BP Jacob A. Role of Complement and Potential of Complement Inhibitors in Myasthenia Gravis and Neuromyelitis Optica Spectrum Disorders: A Brief Review. J Neurol (2021) 268:1643–64. doi: 10.1007/s00415-019-09498-4 31482201

[29] Toyka K Brachman D Pestronk A Kao I. Myasthenia Gravis: Passive Transfer From Man to Mouse. Science (1975) 190(4212):397–9. doi: 10.1126/science.1179220 1179220

[30] Bennett JL Lam C Kalluri SR Saikali P Bautista K Dupree C. Intrathecal Pathogenic Anti-Aquaporin-4 Antibodies in Early Neuromyelitis Optica. Ann Neurol (2009) 66(5):617–29. doi: 10.1002/ana.21802 PMC3180961 19938104

[31] Braczynski AK Schulz JB Bach J-P. Vaccination Strategies in Tauopathies and Synucleinopathies. J Neurochem (2017) 143(5):467–88. doi: 10.1111/jnc.14207 28869766

[32] Foltynie T Langston JW. Therapies to Slow, Stop, or Reverse Parkinson’s Disease. J Park Dis (2018) 8(s1):S115–21. doi: 10.3233/JPD-181481 PMC6311371 30584162

[33] Birkmayer W Hornykiewicz O. [The L-3,4-Dioxyphenylalanine (DOPA)-Effect in Parkinson-Akinesia]. Wien Klin Wochenschr (1961) 73:787–8. 13869404

[34] Czech H Zeidman LA. Walther Birkmayer, Co-Describer of L-Dopa, and His Nazi Connections: Victim or Perpetrator? J Hist Neurosci (2014) 23(2):160–91. doi: 10.1080/0964704X.2013.865427 24697654

[35] Olanow CW. Levodopa is the Best Symptomatic Therapy for PD: Nothing More, Nothing Less. Movement Disord (2019) 34(6):812–5. doi: 10.1002/mds.27690 30990922

[36] Mogi M Harada M Narabayashi H Inagaki H Minami M Nagatsu T. Interleukin (IL)-1β, IL-2, IL-4, IL-6 and Transforming Growth Factor-α Levels are Elevated in Ventricular Cerebrospinal Fluid in Juvenile Parkinsonism and Parkinson’s Disease. Neurosci Lett (1996) 211(1):13–6. doi: 10.1016/0304-3940(96)12706-3 8809836

[37] Mogi M Harada M Kondo T Riederer P Inagaki H Minami M. Interleukin-1β, Interleukin-6, Epidermal Growth Factor and Transforming Growth Factor-α are Elevated in the Brain From Parkinsonian Patients. Neurosci Lett (1994) 180(2):147–50. doi: 10.1016/0304-3940(94)90508-8 7700568

[38] Mogi M Harada M Riederer P Narabayashi H Fujita K Nagatsu T. Tumor Necrosis Factor-Alpha (TNF-Alpha) Increases Both in the Brain and in the Cerebrospinal Fluid From Parkinsonian Patients. Neurosci Lett (1994) 165(1–2):208–10. doi: 10.1016/0304-3940(94)90746-3 8015728

[39] Qin X-Y Zhang S-P Cao C Loh YP Cheng Y. Aberrations in Peripheral Inflammatory Cytokine Levels in Parkinson Disease: A Systematic Review and Meta-Analysis. JAMA Neurol (2016) 73(11):1316. doi: 10.1001/jamaneurol.2016.2742 27668667

[40] Fan Z Pan Y-T Zhang Z-Y Yang H Yu S-Y Zheng Y. Systemic Activation of NLRP3 Inflammasome and Plasma α-Synuclein Levels Are Correlated With Motor Severity and Progression in Parkinson’s Disease. J Neuroinflamm (2020) 17(1):11. doi: 10.1186/s12974-019-1670-6 PMC6950934 31915018

[41] Sommer A Fadler T Dorfmeister E Hoffmann A-C Xiang W Winner B. Infiltrating T Lymphocytes Reduce Myeloid Phagocytosis Activity in Synucleinopathy Model. J Neuroinflamm (2016) 13(1):174. doi: 10.1186/s12974-016-0632-5 PMC4929755 27364890

[42] Brochard V Combadière B Prigent A Laouar Y Perrin A Beray-Berthat V. Infiltration of CD4+ Lymphocytes Into the Brain Contributes to Neurodegeneration in a Mouse Model of Parkinson Disease. J Clin Invest (2009) 119(1):182–92. doi: 10.1172/JCI36470 PMC2613467 19104149

[43] Stone DK Reynolds AD Mosley RL Gendelman HE. Innate and Adaptive Immunity for the Pathobiology of Parkinson’s Disease. Antioxid Redox Sign (2009) 11(9):2151–66. doi: 10.1089/ars.2009.2460 PMC2788126 19243239

[44] Kustrimovic N Comi C Magistrelli L Rasini E Legnaro M Bombelli R. Parkinson’s Disease Patients Have a Complex Phenotypic and Functional Th1 Bias: Cross-Sectional Studies of CD4+ Th1/Th2/T17 and Treg in Drug-Naïve and Drug-Treated Patients. J Neuroinflamm. (2018) 15(1):205. doi: 10.1186/s12974-018-1248-8 PMC6044047 30001736

[45] Michell-Robinson MA Touil H Healy LM Owen DR Durafourt BA Bar-Or A. Roles of Microglia in Brain Development, Tissue Maintenance and Repair. Brain (2015) 138(5):1138–59. doi: 10.1093/brain/awv066 PMC5963417 25823474

[46] Eyolfson E Khan A Mychasiuk R Lohman AW. Microglia Dynamics in Adolescent Traumatic Brain Injury. J Neuroinflamm (2020) 17(1):326. doi: 10.1186/s12974-020-01994-z PMC7597018 33121516

[47] Cherry JD Tripodis Y Alvarez VE Huber B Kiernan PT Daneshvar DH. Microglial Neuroinflammation Contributes to Tau Accumulation in Chronic Traumatic Encephalopathy. Acta Neuropathol Commun (2016) 4(1):112. doi: 10.1186/s40478-016-0382-8 27793189 PMC5084333

[48] Marques CP Hu S Sheng W Lokensgard JR. Microglial Cells Initiate Vigorous Yet non-Protective Immune Responses During HSV-1 Brain Infection. Virus Res (2006) 121(1):1–10. doi: 10.1016/j.virusres.2006.03.009 16621100

[49] Chapman LM Aggrey AA Field DJ Srivastava K Ture S Yui K. Platelets Present Antigen in the Context of MHC Class I. J Immunol Baltim Md 1950 (2012) 189(2):916–23. doi: 10.4049/jimmunol.1200580 PMC3392496 22706078

[50] Kao K. Plasma and Platelet HLA in Normal Individuals: Quantitation by Competitive Enzyme-Linked Immunoassay. Blood (1987) 70(1):282–6. doi: 10.1182/blood.V70.1.282.282 3593967

[51] Kao K Cook D Scornik J. Quantitative Analysis of Platelet Surface HLA by W6/32 Anti-HLA Monoclonal Antibody. Blood (1986) 68(3):627–32. doi: 10.1182/blood.V68.3.627.627 3742047

[52] Zufferey A Schvartz D Nolli S Reny J-L Sanchez J-C Fontana P. Characterization of the Platelet Granule Proteome: Evidence of the Presence of MHC1 in Alpha-Granules. J Proteomics (2014) 101:130–40. doi: 10.1016/j.jprot.2014.02.008 24549006

[53] Panicker N Sarkar S Harischandra DS Neal M Kam T-I Jin H. Fyn Kinase Regulates Misfolded α-Synuclein Uptake and NLRP3 Inflammasome Activation in Microgliafyn Mediates NLRP3 Inflammasome Activation in PD. J Exp Med (2019) 216(6):1411–30. doi: 10.1084/jem.20182191 PMC6547864 31036561

[54] Ferreira SA Romero-Ramos M. Microglia Response During Parkinson’s Disease: Alpha-Synuclein Intervention. Front Cell Neurosci (2018) 12:247. doi: 10.3389/fncel.2018.00247 30127724 PMC6087878

[55] Colonna M Butovsky O. Microglia Function in the Central Nervous System During Health and Neurodegeneration. Annu Rev Immunol (2017) 35(1):441–68. doi: 10.1146/annurev-immunol-051116-052358 PMC8167938 28226226

[56] Prinz M Priller J. The Role of Peripheral Immune Cells in the CNS in Steady State and Disease. Nat Neurosci (2017) 20(2):136–44. doi: 10.1038/nn.4475 28092660

[57] Srinivasan K Friedman BA Larson JL Lauffer BE Goldstein LD Appling LL. Untangling the Brain’s Neuroinflammatory and Neurodegenerative Transcriptional Responses. Nat Commun (2016) 7(1):11295. doi: 10.1038/ncomms11295 27097852 PMC4844685

[58] Chen X Hu Y Cao Z Liu Q Cheng Y. Cerebrospinal Fluid Inflammatory Cytokine Aberrations in Alzheimer’s Disease, Parkinson’s Disease and Amyotrophic Lateral Sclerosis: A Systematic Review and Meta-Analysis. Front Immunol (2018) 9:2122. doi: 10.3389/fimmu.2018.02122 30283455 PMC6156158

[59] Ouchi Y Yoshikawa E Sekine Y Futatsubashi M Kanno T Ogusu T. Microglial Activation and Dopamine Terminal Loss in Early Parkinson’s Disease. Ann Neurol (2005) 57(2):168–75. doi: 10.1002/ana.20338 15668962

[60] Ouchi Y Yagi S Yokokura M Sakamoto M. Neuroinflammation in the Living Brain of Parkinson’s Disease. Parkinsonism Relat D (2009) 15:S200–4. doi: 10.1016/S1353-8020(09)70814-4 20082990

[61] Stokholm MG Iranzo A Østergaard K Serradell M Otto M Svendsen KB. Assessment of Neuroinflammation in Patients With Idiopathic Rapid-Eye-Movement Sleep Behaviour Disorder: A Case-Control Study. Lancet Neurol (2017) 16(10):789–96. doi: 10.1016/S1474-4422(17)30173-4 28684245

[62] Earls RH Menees KB Chung J Barber J Gutekunst C-A Hazim MG. Intrastriatal Injection of Preformed Alpha-Synuclein Fibrils Alters Central and Peripheral Immune Cell Profiles in Non-Transgenic Mice. J Neuroinflamm (2019) 16(1):250. doi: 10.1186/s12974-019-1636-8 PMC6889316 31796095

[63] Kuhbandner K Hoffmann A Alvarado MNG Seyler L Bäuerle T Winkler J. Alpha-Synuclein: A Modulator During Inflammatory CNS Demyelination. J Mol Neurosci (2020) 70:1038–49. doi: 10.1007/s12031-020-01498-8 PMC7334286 32207050

[64] Dinarello CA. Overview of the IL-1 Family in Innate Inflammation and Acquired Immunity. Immunol Rev (2017) 281(1):8–27. doi: 10.1111/imr.12621 PMC5756628 29247995

[65] McGeer PL McGeer EG. Inflammation and the Degenerative Diseases of Aging. Ann Ny Acad Sci (2004) 1035(1):104–16. doi: 10.1196/annals.1332.007 15681803

[66] Hirsch EC Standaert DG. Ten Unsolved Questions About Neuroinflammation in Parkinson’s Disease. Mov Disord Off J Mov Disord Soc (2021) 36(1):16–24. doi: 10.1002/mds.28075 32357266

[67] Pouchieu C Piel C Carles C Gruber A Helmer C Tual S. Pesticide Use in Agriculture and Parkinson’s Disease in the AGRICAN Cohort Study. Int J Epidemiol (2018) 47(1):299–310. doi: 10.1093/ije/dyx225 29136149

[68] Fazzini E Fleming J Fahn S. Cerebrospinal Fluid Antibodies to Coronavirus in Patients With Parkinson’s Disease. Movement Disord (1992) 7(2):153–8. doi: 10.1002/mds.870070210 PMC7168426 1316552

[69] Netland J Meyerholz DK Moore S Cassell M Perlman S. Severe Acute Respiratory Syndrome Coronavirus Infection Causes Neuronal Death in the Absence of Encephalitis in Mice Transgenic for Human ACE2. J Virol (2008) 82(15):7264–75. doi: 10.1128/JVI.00737-08 PMC2493326 18495771

[70] Semerdzhiev SA Fakhree MAA Segers-Nolten I Blum C Claessens MMAE. Interactions Between SARS-CoV-2 N-Protein and α-Synuclein Accelerate Amyloid Formation. ACS Chem Neurosci (2022) 13:143–50. doi: 10.1101/2021.04.12.439549 PMC8739828 34860005

[71] Brundin P Nath A Beckham JD. Is COVID-19 a Perfect Storm for Parkinson’s Disease? Trends Neurosci (2020) 43(12):931–3. doi: 10.1016/j.tins.2020.10.009 PMC7577682 33158605

[72] Makhoul K Jankovic J. Parkinson’s Disease After COVID-19. J Neurol Sci (2021) 422:117331. doi: 10.1016/j.jns.2021.117331 33540185 PMC9755718

[73] Sommer A Maxreiter F Krach F Fadler T Grosch J Maroni M. Th17 Lymphocytes Induce Neuronal Cell Death in a Human iPSC-Based Model of Parkinson’s Disease. Cell Stem Cell (2018) 23(1):123–31.e6. doi: 10.1016/j.stem.2018.06.015 29979986

[74] Galiano-Landeira J Torra A Vila M Bové J. CD8 T Cell Nigral Infiltration Precedes Synucleinopathy in Early Stages of Parkinson’s Disease. Brain (2020) 143(12):3717–33. doi: 10.1093/brain/awaa269 33118032

[75] Karikari AA McFleder RL Ribechini E Blum R Bruttel V Knorr S. Neurodegeneration by α-Synuclein-Specific T Cells in AAV-A53T-α-Synuclein Parkinson’s Disease Mice. Brain Behav Immun (2022) 101:194–210. doi: 10.1016/j.bbi.2022.01.007 35032575

[76] Bas J Calopa M Mestre M Molleví DG Cutillas B Ambrosio S. Lymphocyte Populations in Parkinson’s Disease and in Rat Models of Parkinsonism. J Neuroimmunol (2001) 113(1):146–52. doi: 10.1016/S0165-5728(00)00422-7 11137586

[77] Saunders JAH Estes KA Kosloski LM Allen HE Dempsey KM Torres-Russotto DR. CD4+ Regulatory and Effector/Memory T Cell Subsets Profile Motor Dysfunction in Parkinson’s Disease. J Neuroimmune Pharm (2012) 7(4):927–38. doi: 10.1007/s11481-012-9402-z PMC3515774 23054369

[78] Jiang S Gao H Luo Q Wang P Yang X. The Correlation of Lymphocyte Subsets, Natural Killer Cell, and Parkinson’s Disease: A Meta-Analysis. Neurol Sci (2017) 38(8):1373–80. doi: 10.1007/s10072-017-2988-4 28497309

[79] Baba Y Kuroiwa A Uitti RJ Wszolek ZK Yamada T. Alterations of T-Lymphocyte Populations in Parkinson Disease. Parkinsonism Relat D (2005) 11(8):493–8. doi: 10.1016/j.parkreldis.2005.07.005 16154792

[80] Rosenkranz D Weyer S Tolosa E Gaenslen A Berg D Leyhe T. Higher Frequency of Regulatory T Cells in the Elderly and Increased Suppressive Activity in Neurodegeneration. J Neuroimmunol (2007) 188(1–2):117–27. doi: 10.1016/j.jneuroim.2007.05.011 17582512

[81] Chen Y Qi B Xu W Ma B Li L Chen Q. Clinical Correlation of Peripheral CD4+-Cell Sub-Sets, Their Imbalance and Parkinson’s Disease. Mol Med Rep (2015) 12(4):6105–11. doi: 10.3892/mmr.2015.4136 26239429

[82] McGeer PL Itagaki S Akiyama H McGeer EG. Rate of Cell Death in Parkinsonism Indicates Active Neuropathological Process. Ann Neurol (1988) 24(4):574–6. doi: 10.1002/ana.410240415 3239957

[83] Chen S Liu Y Niu Y Xu Y Zhou Q Xu X. Increased Abundance of Myeloid-Derived Suppressor Cells and Th17 Cells in Peripheral Blood of Newly-Diagnosed Parkinson’s Disease Patients. Neurosci Lett (2017) 648:21–5. doi: 10.1016/j.neulet.2017.03.045 28359932

[84] Chen Y Yu M Liu X Qu H Chen Q Qian W. Clinical Characteristics and Peripheral T Cell Subsets in Parkinson’s Disease Patients With Constipation. Int J Clin Exp Patho (2015) 8(3):2495–504. PMC4440064 26045755

[85] Storelli E Cassina N Rasini E Marino F Cosentino M. Do Th17 Lymphocytes and IL-17 Contribute to Parkinson’s Disease? A Systematic Review of Available Evidence. Front Neurol (2019) 10:13. doi: 10.3389/fneur.2019.00013 30733703 PMC6353825

[86] Shukla AW Fox SH. Th17 Lymphocyte Spearheads the Immune Attack in Parkinson’s Disease: New Evidence for Neuronal Death. Movement Disord (2018) 33(10):1560–0. doi: 10.1002/mds.27496 PMC6214624 30365216

[87] Raj T Rothamel K Mostafavi S Ye C Lee MN Replogle JM. Polarization of the Effects of Autoimmune and Neurodegenerative Risk Alleles in Leukocytes. Sci New York NY (2014) 344(6183):519–23. doi: 10.1126/science.1249547 PMC4910825 24786080

[88] Garretti F Agalliu D Arlehamn CSL Sette A Sulzer D. Autoimmunity in Parkinson’s Disease: The Role of α-Synuclein-Specific T Cells. Front Immunol (2019) 10:303. doi: 10.3389/fimmu.2019.00303 30858851 PMC6397885

[89] Dhanwani R Lima-Junior JR Sethi A Pham J Williams G Frazier A. Transcriptional Analysis of Peripheral Memory T Cells Reveals Parkinson’s Disease-Specific Gene Signatures. NPJ Park Dis (2022) 8(1):30. doi: 10.1038/s41531-022-00282-2 PMC8938520 35314697

[90] de Graaf MT Smitt PAES Luitwieler RL van Velzen C van den Broek PDM Kraan J. Central Memory CD4+ T Cells Dominate the Normal Cerebrospinal Fluid. Cytom Part B Clin Cytom (2011) 80B(1):43–50. doi: 10.1002/cyto.b.20542 20632412

[91] Hong S Zhang Z Liu H Tian M Zhu X Zhang Z. B Cells Are the Dominant Antigen-Presenting Cells That Activate Naive CD4+ T Cells Upon Immunization With a Virus-Derived Nanoparticle Antigen. Immunity (2018) 49(4):695–708.e4. doi: 10.1016/j.immuni.2018.08.012 30291027

[92] Bell L Lenhart A Rosenwald A Monoranu CM Berberich-Siebelt F. Lymphoid Aggregates in the CNS of Progressive Multiple Sclerosis Patients Lack Regulatory T Cells. Front Immunol (2020) 10:3090. doi: 10.3389/fimmu.2019.03090 32010141 PMC6974514

[93] Rowe DB Le W Smith RG Appel SH. Antibodies From Patients With Parkinson’s Disease React With Protein Modified by Dopamine Oxidation. J Neurosci Res (1998) 53(5):551–8. doi: 10.1002/(SICI)1097-4547(19980901)53:5<551::AID-JNR5>3.0.CO;2-8 9726426

[94] Orr CF Rowe DB Mizuno Y Mori H Halliday GM. A Possible Role for Humoral Immunity in the Pathogenesis of Parkinson’s Disease. Brain (2005) 128(11):2665–74. doi: 10.1093/brain/awh625 16219675

[95] Han M Nagele E DeMarshall C Acharya N Nagele R. Diagnosis of Parkinson’s Disease Based on Disease-Specific Autoantibody Profiles in Human Sera. PLoS One (2012) 7(2):e32383. doi: 10.1371/journal.pone.0032383 22384236 PMC3285212

[96] Li R Tropea TF Baratta LR Zuroff L Diaz-Ortiz ME Zhang B. Abnormal B-Cell and Tfh-Cell Profiles in Patients With Parkinson Disease: A Cross-Sectional Study. Neurol - Neuroimmunol Neuroinflamm (2022) 9(2):e1125. doi: 10.1212/NXI.0000000000001125 34955458 PMC8711073

[97] Sun C Yu W Zhao Z Song C Liu Y Jia G. Peripheral Humoral Immune Response Is Associated With the Non-Motor Symptoms of Parkinson’s Disease. Front Neurosci-switz (2019) 13:1057. doi: 10.3389/fnins.2019.01057 PMC6795918 31649497

[98] Wang P Luo M Zhou W Jin X Xu Z Yan S. Global Characterization of Peripheral B Cells in Parkinson’s Disease by Single-Cell RNA and BCR Sequencing. Front Immunol (2022) 13:814239. doi: 10.3389/fimmu.2022.814239 35250991 PMC8888848

[99] Li X Koudstaal W Fletcher L Costa M van Winsen M Siregar B. Naturally Occurring Antibodies Isolated From PD Patients Inhibit Synuclein Seeding *In Vitro* and Recognize Lewy Pathology. Acta Neuropathol (2019) 137(5):825–36. doi: 10.1007/s00401-019-01974-5 PMC6482120 30805666

[100] Shalash A Salama M Makar M Roushdy T Elrassas HH Mohamed W. Elevated Serum α-Synuclein Autoantibodies in Patients With Parkinson’s Disease Relative to Alzheimer’s Disease and Controls. Front Neurol (2017) 8:720. doi: 10.3389/fneur.2017.00720 29312137 PMC5744443

[101] Ray A Basu S Williams CB Salzman NH Dittel BN. A Novel IL-10–Independent Regulatory Role for B Cells in Suppressing Autoimmunity by Maintenance of Regulatory T Cells *via* GITR Ligand. J Immunol (2012) 188(7):3188–98. doi: 10.4049/jimmunol.1103354 PMC3311743 22368274

[102] Hoffman PM Robbins DS Oldstone MBA Gibbs CJ Gajdusek DC. Humoral Immunity in Guamanians With Amyotrophic Lateral Sclerosis and Parkinsonism-Dementia. Ann Neurol (1981) 10(2):193–6. doi: 10.1002/ana.410100210 7283404

[103] Sabatino JJ Pröbstel A-K Zamvil SS. B Cells in Autoimmune and Neurodegenerative Central Nervous System Diseases. Nat Rev Neurosci (2019) 20(12):728–45. doi: 10.1038/s41583-019-0233-2 31712781

[104] Orsini F Blasio DD Zangari R Zanier ER Simoni M-GD. Versatility of the Complement System in Neuroinflammation, Neurodegeneration and Brain Homeostasis. Front Cell Neurosci (2014) 8:380. doi: 10.3389/fncel.2014.00380 25426028 PMC4224073

[105] Ahn JJ Abu-Rub M Miller RH. B Cells in Neuroinflammation: New Perspectives and Mechanistic Insights. Cells (2021) 10(7):1605. doi: 10.3390/cells10071605 34206848 PMC8305155

[106] Racette BA Gross A Vouri SM Camacho-Soto A Willis AW Nielsen SS. Immunosuppressants and Risk of Parkinson Disease. Ann Clin Transl Neur. (2018) 5(7):870–5. doi: 10.1002/acn3.580 PMC6043771 30009205

[107] Hancock DB Martin ER Stajich JM Jewett R Stacy MA Scott BL. Smoking, Caffeine, and Nonsteroidal Anti-Inflammatory Drugs in Families With Parkinson Disease. Arch Neurol-Chicago (2007) 64(4):576. doi: 10.1001/archneur.64.4.576 17420321

[108] Qiu F Liang C-L Liu H Zeng Y-Q Hou S Huang S. Impacts of Cigarette Smoking on Immune Responsiveness: Up and Down or Upside Down? Oncotarget (2016) 8(1):268–84. doi: 10.18632/oncotarget.13613 PMC5352117 27902485

[109] Sopori M. Effects of Cigarette Smoke on the Immune System. Nat Rev Immunol (2002) 2(5):372–7. doi: 10.1038/nri803 12033743

[110] Ansel H David M Yiyun H Nabeel N Jim R Richard C. Imaging Dynamic Neuroimmune Responses to LPS in Tobacco Smokers: A [11C]PBR28 PET Study. J Nucl Med (2019) 60(Suppl 1):488.

[111] Fan J Nielsen SS Faust IM Racette BA. Transplant and Risk of Parkinson Disease. Parkinsonism Relat D (2019) 63:149–55. doi: 10.1016/j.parkreldis.2019.02.013 30827837

[112] Peter I Dubinsky M Bressman S Park A Lu C Chen N. Anti-Tumor Necrosis Factor Therapy and Incidence of Parkinson Disease Among Patients With Inflammatory Bowel Disease. JAMA Neurol (2018) 75(8):939. doi: 10.1001/jamaneurol.2018.0605 29710331 PMC6142934

[113] Camacho-Soto A Gross A Nielsen SS Dey N Racette BA. Inflammatory Bowel Disease and Risk of Parkinson’s Disease in Medicare Beneficiaries. Parkinsonism Relat D (2018) 50:23–8. doi: 10.1016/j.parkreldis.2018.02.008 PMC5943158 29459115

[114] Chen H Jacobs E Schwarzschild MA McCullough ML Calle EE Thun MJ. Nonsteroidal Antiinflammatory Drug Use and the Risk for Parkinson’s Disease. Ann Neurol (2005) 58(6):963–7. doi: 10.1002/ana.20682 16240369

[115] Gao X Chen H Schwarzschild MA Ascherio A. Use of Ibuprofen and Risk of Parkinson Disease(E–Pub Ahead of Print). Neurology (2011) 76(10):863–9. doi: 10.1212/WNL.0b013e31820f2d79 PMC3059148 21368281

[116] Asanuma M Nishibayashi-Asanuma S Miyazaki I Kohno M Ogawa N. Neuroprotective Effects of non-Steroidal Anti-Inflammatory Drugs by Direct Scavenging of Nitric Oxide Radicals. J Neurochem (2001) 76(6):1895–904. doi: 10.1046/j.1471-4159.2001.00205.x 11259508

[117] Casper D Yaparpalvi U Rempel N Werner P. Ibuprofen Protects Dopaminergic Neurons Against Glutamate Toxicity In Vitro. Neurosci Lett (2000) 289(3):201–4. doi: 10.1016/S0304-3940(00)01294-5 10961664

[118] Bower JH Maraganore DM Peterson BJ Ahlskog JE Rocca WA. Immunologic Diseases, Anti-Inflammatory Drugs, and Parkinson Disease: A Case-Control Study. Neurology (2006) 67(3):494–6. doi: 10.1212/01.wnl.0000227906.99570.cc 16894114

[119] Fang L Karakiulakis G Roth M. Are Patients With Hypertension and Diabetes Mellitus at Increased Risk for COVID-19 Infection? Lancet Respir Med (2020) 8(4):e21. doi: 10.1016/S2213-2600(20)30116-8 32171062 PMC7118626

[120] Qiao W Wang C Chen B Zhang F Liu Y Lu Q. Ibuprofen Attenuates Cardiac Fibrosis in Streptozotocin-Induced Diabetic Rats. Cardiology (2015) 131(2):97–106. doi: 10.1159/000375362 25896805

[121] Hopfner F Höglinger GU Kuhlenbäumer G Pottegård A Wod M Christensen K. β-Adrenoreceptors and the Risk of Parkinson’s Disease. Lancet Neurol (2020) 19(3):247–54. doi: 10.1016/S1474-4422(19)30400-4 31999942

[122] Magistrelli L Comi C. Beta2-Adrenoceptor Agonists in Parkinson’s Disease and Other Synucleinopathies. J Neuroimmune Pharmacol (2020) 15(1):74–81. doi: 10.1007/s11481-018-09831-0 30617750

[123] Perez-Lloret S Otero-Losada M Toblli JE Capani F. Renin-Angiotensin System as a Potential Target for New Therapeutic Approaches in Parkinson’s Disease. Expert Opin Inv Drug (2017) 26(10):1163–73. doi: 10.1080/13543784.2017.1371133 28836869

[124] Jeong S Jang W Shin DW. Association of Statin Use With Parkinson’s Disease: Dose–response Relationship. Movement Disord (2019) 34(7):1014–21. doi: 10.1002/mds.27681 30938893

[125] Samii A Carleton BC Etminan M. Statin Use and the Risk of Parkinson Disease: A Nested Case Control Study. J Clin Neurosci Off J Neurosurg Soc Australas (2008) 15(11):1272–3. doi: 10.1016/j.jocn.2008.01.016 18823780

[126] Poly TN Islam MM Walther BA Yang H-C Nguyen P-A Huang C-W. Exploring the Association Between Statin Use and the Risk of Parkinson’s Disease: A Meta-Analysis of Observational Studies. Neuroepidemiology (2017) 49(3–4):142–51. doi: 10.1159/000480401 29145202

[127] Shi Q Liu S Fonseca VA Thethi TK Shi L. Effect of Metformin on Neurodegenerative Disease Among Elderly Adult US Veterans With Type 2 Diabetes Mellitus. BMJ Open (2019) 9(7):e024954. doi: 10.1136/bmjopen-2018-024954 PMC6677947 31366635

[128] Kuan Y-C Huang K-W Lin C-L Hu C-J Kao C-H. Effects of Metformin Exposure on Neurodegenerative Diseases in Elderly Patients With Type 2 Diabetes Mellitus. Prog Neuro-Psychoph (2017) 79(Pt B):77–83. doi: 10.1016/j.pnpbp.2017.06.002 28583443

[129] Athauda D Foltynie T. Insulin Resistance and Parkinson’s Disease: A New Target for Disease Modification? Prog Neurobiol (2016) 145–146:98–120. doi: 10.1016/j.pneurobio.2016.10.001 27713036

[130] Aviles-Olmos I Limousin P Lees A Foltynie T. Parkinson’s Disease, Insulin Resistance and Novel Agents of Neuroprotection. Brain (2013) 136(Pt 2):374–84. doi: 10.1093/brain/aws009 22344583

[131] Yang L Wang H Liu L Xie A. The Role of Insulin/IGF-1/PI3K/Akt/Gsk3β Signaling in Parkinson’s Disease Dementia. Front Neurosci-switz (2018) 12:73. doi: 10.3389/fnins.2018.00073 PMC5826217 29515352

[132] During MJ Cao L Zuzga DS Francis JS Fitzsimons HL Jiao X. Glucagon-Like Peptide-1 Receptor is Involved in Learning and Neuroprotection. Nat Med (2003) 9(9):1173–9. doi: 10.1038/nm919 12925848

[133] Chen S Yu S-J Li Y Lecca D Glotfelty E Kim HK. Post-Treatment With PT302, a Long-Acting Exendin-4 Sustained Release Formulation, Reduces Dopaminergic Neurodegeneration in a 6-Hydroxydopamine Rat Model of Parkinson’s Disease. Sci Rep-uk (2018) 8(1):10722. doi: 10.1038/s41598-018-28449-z PMC6048117 30013201

[134] Bertilsson G Patrone C Zachrisson O Andersson A Dannaeus K Heidrich J. Peptide Hormone Exendin-4 Stimulates Subventricular Zone Neurogenesis in the Adult Rodent Brain and Induces Recovery in an Animal Model of Parkinson’s Disease. J Neurosci Res (2008) 86(2):326–38. doi: 10.1002/jnr.21483 17803225

[135] Liu W Li Y Jalewa J Saunders-Wood T Li L Hölscher C. Neuroprotective Effects of an Oxyntomodulin Analogue in the MPTP Mouse Model of Parkinson’s Disease. Eur J Pharmacol (2015) 765:284–90. doi: 10.1016/j.ejphar.2015.08.038 26302060

[136] Yun SP Kam T-I Panicker N Kim S Oh Y Park J-S. Block of A1 Astrocyte Conversion by Microglia is Neuroprotective in Models of Parkinson’s Disease. Nat Med (2018) 24(7):931–8. doi: 10.1038/s41591-018-0051-5 PMC6039259 29892066

[137] Lee C-H Jeon SJ Cho KS Moon E Sapkota A Jun HS. Activation of Glucagon-Like Peptide-1 Receptor Promotes Neuroprotection in Experimental Autoimmune Encephalomyelitis by Reducing Neuroinflammatory Responses. Mol Neurobiol (2018) 55(4):3007–20. doi: 10.1007/s12035-017-0550-2 28456941

[138] Wu H-Y Tang X-Q Mao X-F Wang Y-X. Autocrine Interleukin-10 Mediates Glucagon-Like Peptide-1 Receptor-Induced Spinal Microglial β-Endorphin Expression. J Neurosci (2017) 37(48):11701–14. doi: 10.1523/JNEUROSCI.1799-17.2017 PMC6705741 29084866

[139] National Library of Medicine (U.S.). GLP1R in Parkinson's Disease. (2018) Identifier NCT03659682. clinicaltrials.gov-NCT03659682.

[140] Zhu X Han Y Du J Liu R Jin K Yi W. Microbiota-Gut-Brain Axis and the Central Nervous System. Oncotarget (2017) 8(32):53829–38. doi: 10.18632/oncotarget.17754 PMC5581153 28881854

[141] Bienenstock J Kunze W Forsythe P. Microbiota and the Gut–Brain Axis. Nutr Rev (2015) 73(suppl 1):28–31. doi: 10.1093/nutrit/nuv019 26175487

[142] Wang H-X Wang Y-P. Gut Microbiota-Brain Axis. Chin Med J-peking (2016) 129(19):2373–80. doi: 10.4103/0366-6999.190667 PMC5040025 27647198

[143] Sampson TR Debelius JW Thron T Janssen S Shastri GG Ilhan ZE. Gut Microbiota Regulate Motor Deficits and Neuroinflammation in a Model of Parkinson’s Disease. Cell (2016) 167(6):1469–80.e12. doi: 10.1016/j.cell.2016.11.018 27912057 PMC5718049

[144] Olszak T An D Zeissig S Vera MP Richter J Franke A. Microbial Exposure During Early Life has Persistent Effects on Natural Killer T Cell Function. Sci New York NY (2012) 336(6080):489–93. doi: 10.1126/science.1219328 PMC3437652 22442383

[145] Quigley EMM. Microbiota-Brain-Gut Axis and Neurodegenerative Diseases. Curr Neurol Neurosci (2017) 17(12):94. doi: 10.1007/s11910-017-0802-6 29039142

[146] Bansal V Costantini T Kroll L Peterson C Loomis W Eliceiri B. Traumatic Brain Injury and Intestinal Dysfunction: Uncovering the Neuro-Enteric Axis. J Neurotraum (2009) 26(8):1353–9. doi: 10.1089/neu.2008.0858 PMC2989839 19344293

[147] Katzenberger RJ Chtarbanova S Rimkus SA Fischer JA Kaur G Seppala JM. Death Following Traumatic Brain Injury in Drosophila is Associated With Intestinal Barrier Dysfunction. Elife (2015) 4:e04790. doi: 10.7554/eLife.04790 PMC4377547 25742603

[148] Sun M-F Shen Y-Q. Dysbiosis of Gut Microbiota and Microbial Metabolites in Parkinson’s Disease. Ageing Res Rev (2018) 45:53–61. doi: 10.1016/j.arr.2018.04.004 29705121

[149] Forsyth CB Shannon KM Kordower JH Voigt RM Shaikh M Jaglin JA. Increased Intestinal Permeability Correlates With Sigmoid Mucosa Alpha-Synuclein Staining and Endotoxin Exposure Markers in Early Parkinson’s Disease. PloS One (2011) 6(12):e28032. doi: 10.1371/journal.pone.0028032 22145021 PMC3228722

[150] Braak H de Vos RAI Bohl J Tredici KD. Gastric α-Synuclein Immunoreactive Inclusions in Meissner’s and Auerbach’s Plexuses in Cases Staged for Parkinson’s Disease-Related Brain Pathology. Neurosci Lett (2006) 396(1):67–72. doi: 10.1016/j.neulet.2005.11.012 16330147

[151] Chartier-Harlin M-C Kachergus J Roumier C Mouroux V Douay X Lincoln S. α-Synuclein Locus Duplication as a Cause of Familial Parkinson’s Disease. Lancet (2004) 364(9440):1167–9. doi: 10.1016/S0140-6736(04)17103-1 15451224

[152] Singleton AB Farrer M Johnson J Singleton A Hague S Kachergus J. α-Synuclein Locus Triplication Causes Parkinson9s Disease. Science (2003) 302(5646):841–1. doi: 10.1126/science.1090278 14593171

[153] Jowaed A Schmitt I Kaut O Wüllner U. Methylation Regulates Alpha-Synuclein Expression and Is Decreased in Parkinson’s Disease Patients’ Brains. J Neurosci (2010) 30(18):6355–9. doi: 10.1523/JNEUROSCI.6119-09.2010 PMC6632710 20445061

[154] National Library of Medicine (U.S.). Gut Microbiota and Parkinson's Disease. (2018) Identifier NCT03710668. clinicaltrials.gov-NCT03710668.

[155] Coles LD Tuite PJ Öz G Mishra UR Kartha RV Sullivan KM. Repeated-Dose Oral N-Acetylcysteine in Parkinson’s Disease: Pharmacokinetics and Effect on Brain Glutathione and Oxidative Stress. J Clin Pharmacol (2018) 58(2):158–67. doi: 10.1002/jcph.1008 PMC5762253 28940353

[156] Smeyne M Smeyne RJ. Glutathione Metabolism and Parkinson’s Disease. Free Radic Biol Med (2013) 62:13–25. doi: 10.1016/j.freeradbiomed.2013.05.001 23665395 PMC3736736

[157] Dias V Junn E Mouradian MM. The Role of Oxidative Stress in Parkinson’s Disease. J Park Dis (2013) 3(4):461–91. doi: 10.3233/JPD-130230 PMC4135313 24252804

[158] Giasson BI Duda JE Murray IVJ Chen Q Souza JM Hurtig HI. Oxidative Damage Linked to Neurodegeneration by Selective α-Synuclein Nitration in Synucleinopathy Lesions. Science (2000) 290(5493):985–9. doi: 10.1126/science.290.5493.985 11062131

[159] Andersen JK. Oxidative Stress in Neurodegeneration: Cause or Consequence? Nat Med (2004) 10(Suppl 7):S18–25. doi: 10.1038/nrn1434 15298006

[160] Bandookwala M Sengupta P. 3-Nitrotyrosine: A Versatile Oxidative Stress Biomarker for Major Neurodegenerative Diseases. Int J Neurosci (2020) 130(10):1–16. doi: 10.1080/00207454.2020.1713776 31914343

[161] clinicaltrials.gov-NCT02445651. Physiological Effects of Nutritional Support in Patients With Parkinson’s Disease (201). Available at: https://clinicaltrials.gov/ct2/show/NCT02445651.

[162] Weiss R Schilling E Grahnert A Kölling V Dorow J Ceglarek U. Nicotinamide: A Vitamin Able to Shift Macrophage Differentiation Toward Macrophages With Restricted Inflammatory Features. Innate Immun (2015) 21(8):813–26. doi: 10.1177/1753425915602545 26385774

[163] Jia H Li X Gao H Feng Z Li X Zhao L. High Doses of Nicotinamide Prevent Oxidative Mitochondrial Dysfunction in a Cellular Model and Improve Motor Deficit in a Drosophila Model of Parkinson’s Disease. J Neurosci Res (2008) 86(9):2083–90. doi: 10.1002/jnr.21650 18381761

[164] Wakade C Chong R Bradley E Thomas B Morgan J. Upregulation of GPR109A in Parkinson’s Disease. PLoS One (2014) 9(10):e109818. doi: 10.1371/journal.pone.0109818 25329911 PMC4201464

[165] Giri B Belanger K Seamon M Bradley E Purohit S Chong R. Niacin Ameliorates Neuro-Inflammation in Parkinson’s Disease *via* GPR109A. Int J Mol Sci (2019) 20(18):4559. doi: 10.3390/ijms20184559 PMC6770365 31540057

[166] Seamon M Purohit S Giri B Baban B Morgan J Chong R. Niacin for Parkinson’s Disease. Clin Exp Neuroimmunol (2019) 11(1):47–56. doi: 10.1111/cen3.12553

[167] clinicaltrials.gov-NCT03808961. Niacin for Parkinsons Disease (2019). Available at: https://clinicaltrials.gov/ct2/show/NCT03808961.

[168] Ungerstedt U. 6-Hydroxy-Dopamine Induced Degeneration of Central Monoamine Neurons. Eur J Pharmacol (1968) 5(1):107–10. doi: 10.1016/0014-2999(68)90164-7 5718510

[169] Langston JW. MPTP And Parkinson’s Disease. Trends Neurosci (1985) 8:79–83. doi: 10.1016/0166-2236(85)90031-1

[170] Parillaud VR Lornet G Monnet Y Privat A-L Haddad AT Brochard V. Analysis of Monocyte Infiltration in MPTP Mice Reveals That Microglial CX3CR1 Protects Against Neurotoxic Over-Induction of Monocyte-Attracting CCL2 by Astrocytes. J Neuroinflamm. (2017) 14(1):60. doi: 10.1186/s12974-017-0830-9 PMC5359822 28320442

[171] Lee E Hwang I Park S Hong S Hwang B Cho Y. MPTP-Driven NLRP3 Inflammasome Activation in Microglia Plays a Central Role in Dopaminergic Neurodegeneration. Cell Death Differ (2018) 26(2):213–28. doi: 10.1038/s41418-018-0124-5 PMC6329843 29786072

[172] Simola N Morelli M Carta AR. The 6-Hydroxydopamine Model of Parkinson’s Disease. Neurotox Res (2007) 11(3–4):151–67. doi: 10.1007/BF03033565 17449457

[173] McGeer PL Kawamata T Walker DG Akiyama H Tooyama I McGeer EG. Microglia in Degenerative Neurological Disease. Glia (1993) 7(1):84–92. doi: 10.1002/glia.440070114 8423066

[174] Ambrosi G Kustrimovic N Siani F Rasini E Cerri S Ghezzi C. Complex Changes in the Innate and Adaptive Immunity Accompany Progressive Degeneration of the Nigrostriatal Pathway Induced by Intrastriatal Injection of 6-Hydroxydopamine in the Rat. Neurotox Res (2017) 32(1):71–81. doi: 10.1007/s12640-017-9712-2 28285346

[175] Singh SS Rai SN Birla H Zahra W Rathore AS Singh SP. NF-κb-Mediated Neuroinflammation in Parkinson’s Disease and Potential Therapeutic Effect of Polyphenols. Neurotox Res (2019) 37(3):491–507. doi: 10.1007/s12640-019-00147-2 31823227

[176] Tang Y Le W. Differential Roles of M1 and M2 Microglia in Neurodegenerative Diseases. Mol Neurobiol (2015) 53(2):1181–94. doi: 10.1007/s12035-014-9070-5 25598354

[177] Hunot S Boissière F Faucheux B Brugg B Mouatt-Prigent A Agid Y. Nitric Oxide Synthase and Neuronal Vulnerability in Parkinson’s Disease. Neuroscience (1996) 72(2):355–63. doi: 10.1016/0306-4522(95)00578-1 8737406

[178] Ghosh A Roy A Liu X Kordower JH Mufson EJ Hartley DM. Selective Inhibition of NF-κb Activation Prevents Dopaminergic Neuronal Loss in a Mouse Model of Parkinson’s Disease. Proc Natl Acad Sci (2007) 104(47):18754–9. doi: 10.1073/pnas.0704908104 PMC2141849 18000063

[179] Noelker C Morel L Lescot T Osterloh A Alvarez-Fischer D Breloer M. Toll Like Receptor 4 Mediates Cell Death in a Mouse MPTP Model of Parkinson Disease. Sci Rep-uk (2013) 3(1):1393. doi: 10.1038/srep01393 PMC3589722 23462811

[180] Qureshi ST Larivière L Leveque G Clermont S Moore KJ Gros P. Endotoxin-Tolerant Mice Have Mutations in Toll-Like Receptor 4 (Tlr4). J Exp Med (1999) 189(4):615–25. doi: 10.1084/jem.189.4.615 PMC2192941 9989976

[181] Mao Z Liu C Ji S Yang Q Ye H Han H. The NLRP3 Inflammasome is Involved in the Pathogenesis of Parkinson’s Disease in Rats. Neurochem Res (2017) 42(4):1104–15. doi: 10.1007/s11064-017-2185-0 28247334

[182] Heng Y Zhang Q-S Mu Z Hu J-F Yuan Y-H Chen N-H. Ginsenoside Rg1 Attenuates Motor Impairment and Neuroinflammation in the MPTP-Probenecid-Induced Parkinsonism Mouse Model by Targeting α-Synuclein Abnormalities in the Substantia Nigra. Toxicol Lett (2016) 243:7–21. doi: 10.1016/j.toxlet.2015.12.005 26723869

[183] Liu J-Q Zhao M Zhang Z Cui L-Y Zhou X Zhang W. Rg1 Improves LPS-Induced Parkinsonian Symptoms in Mice *via* Inhibition of NF-κb Signaling and Modulation of M1/M2 Polarization. Acta Pharmacol Sin (2020) 41(4):523–34. doi: 10.1038/s41401-020-0358-x PMC7468333 32203085

[184] Matheoud D Sugiura A Bellemare-Pelletier A Laplante A Rondeau C Chemali M. Parkinson’s Disease-Related Proteins PINK1 and Parkin Repress Mitochondrial Antigen Presentation. Cell (2016) 166(2):314–27. doi: 10.1016/j.cell.2016.05.039 27345367

[185] Matheoud D Cannon T Voisin A Penttinen A-M Ramet L Fahmy AM. Intestinal Infection Triggers Parkinson’s Disease-Like Symptoms in Pink1–/– Mice. Nature (2019) 571(7766):565–9. doi: 10.1038/s41586-019-1405-y 31316206

[186] Huang B Wu S Wang Z Ge L Rizak JD Wu J. Phosphorylated α-Synuclein Accumulations and Lewy Body-Like Pathology Distributed in Parkinson’s Disease-Related Brain Areas of Aged Rhesus Monkeys Treated With MPTP. Neuroscience (2018) 379:302–15. doi: 10.1016/j.neuroscience.2018.03.026 29592843

[187] Ip CW Klaus L-C Karikari AA Visanji NP Brotchie JM Lang AE. AAV1/2-Induced Overexpression of A53T-α-Synuclein in the Substantia Nigra Results in Degeneration of the Nigrostriatal System With Lewy-Like Pathology and Motor Impairment: A New Mouse Model for Parkinson’s Disease. Acta Neuropathol Commun (2017) 5(1):11. doi: 10.1186/s40478-017-0416-x 28143577 PMC5286802

[188] Haque ME Akther M Jakaria M Kim I Azam S Choi D. Targeting the Microglial NLRP3 Inflammasome and Its Role in Parkinson’s Disease. Mov Disord (2020) 35(1):20–33. doi: 10.1002/mds.27874 31680318

[189] Labzin LI Heneka MT Latz E. Innate Immunity and Neurodegeneration. Annu Rev Med (2018) 69(1):437–49. doi: 10.1146/annurev-med-050715-104343 29106805

[190] Béraud D Twomey M Bloom B Mittereder A Ton V Neitzke K. α-Synuclein Alters Toll-Like Receptor Expression. Front Neurosci-switz (2011) 5:80. doi: 10.3389/fnins.2011.00080 PMC3128248 21747756

[191] Cao S Theodore S Standaert DG. Fcγ Receptors Are Required for NF-κb Signaling, Microglial Activation and Dopaminergic Neurodegeneration in an AAV-Synuclein Mouse Model of Parkinson’s Disease. Mol Neurodegener (2010) 5(1):42. doi: 10.1186/1750-1326-5-42 20977765 PMC2975641

[192] Daëron M. Fc RECEPTOR BIOLOGY. Annu Rev Immunol (1997) 15(1):203–34. doi: 10.1146/annurev.immunol.15.1.203 9143687

[193] Rostami J Fotaki G Sirois J Mzezewa R Bergström J Essand M. Astrocytes Have the Capacity to Act as Antigen-Presenting Cells in the Parkinson’s Disease Brain. J Neuroinflamm. (2020) 17(1):119. doi: 10.1186/s12974-020-01776-7 PMC7164247 32299492

[194] Bido S Muggeo S Massimino L Marzi MJ Giannelli SG Melacini E. Microglia-Specific Overexpression of α-Synuclein Leads to Severe Dopaminergic Neurodegeneration by Phagocytic Exhaustion and Oxidative Toxicity. Nat Commun (2021) 12(1):6237. doi: 10.1038/s41467-021-26519-x 34716339 PMC8556263

[195] Harms AS Cao S Rowse AL Thome AD Li X Mangieri LR. MHCII Is Required for -Synuclein-Induced Activation of Microglia, CD4 T Cell Proliferation, and Dopaminergic Neurodegeneration. J Neurosci (2013) 33(23):9592–600. doi: 10.1523/JNEUROSCI.5610-12.2013 PMC3903980 23739956

[196] Spielman LJ Gibson DL Klegeris A. Unhealthy Gut, Unhealthy Brain: The Role of the Intestinal Microbiota in Neurodegenerative Diseases. Neurochem Int (2018) 120:149–63. doi: 10.1016/j.neuint.2018.08.005 30114473

[197] Schonhoff AM Williams GP Wallen ZD Standaert DG Harms AS. Innate and Adaptive Immune Responses in Parkinson’s Disease. Prog Brain Res (2020) 252:169–216. doi: 10.1016/bs.pbr.2019.10.006 32247364 PMC7185735

[198] Süß P Kalinichenko L Baum W Reichel M Kornhuber J Loskarn S. Hippocampal Structure and Function are Maintained Despite Severe Innate Peripheral Inflammation. Brain Behav Immun (2015) 49:156–70. doi: 10.1016/j.bbi.2015.05.011 26074461

[199] Süß P Hoffmann A Rothe T Ouyang Z Baum W Staszewski O. Chronic Peripheral Inflammation Causes a Region-Specific Myeloid Response in the Central Nervous System. Cell Rep (2020) 30(12):4082–95.e6. doi: 10.1016/j.celrep.2020.02.109 32209470

[200] Arlehamn CSL Dhanwani R Pham J Kuan R Frazier A Dutra JR. α-Synuclein-Specific T Cell Reactivity Is Associated With Preclinical and Early Parkinson’s Disease. Nat Commun (2020) 11(1):1875. doi: 10.1038/s41467-020-15626-w 32313102 PMC7171193

[201] Ip CW Beck SK Volkmann J. Lymphocytes Reduce Nigrostriatal Deficits in the 6-Hydroxydopamine Mouse Model of Parkinson’s Disease. J Neural Transm (2015) 122(12):1633–43. doi: 10.1007/s00702-015-1444-y 26290125

[202] Teismann P. COX-2 in the Neurodegenerative Process of Parkinson’s Disease. Biofactors (2012) 38(6):395–7. doi: 10.1002/biof.1035 PMC3563218 22826171

[203] Chandra G Roy A Rangasamy SB Pahan K. Induction of Adaptive Immunity Leads to Nigrostriatal Disease Progression in MPTP Mouse Model of Parkinson’s Disease. J Immunol (2017) 198(11):4312–26. doi: 10.4049/jimmunol.1700149 PMC5467696 28446566

[204] Chandra G Rangasamy SB Roy A Kordower JH Pahan K. Neutralization of RANTES and Eotaxin Prevents the Loss of Dopaminergic Neurons in a Mouse Model of Parkinson Disease. J Biol Chem (2016) 291(29):15267–81. doi: 10.1074/jbc.M116.714824 PMC4946939 27226559

[205] Roy A Mondal S Kordower JH Pahan K. Attenuation of Microglial RANTES by NEMO-Binding Domain Peptide Inhibits the Infiltration of CD8+ T Cells in the Nigra of Hemiparkinsonian Monkey. Neuroscience (2015) 302:36–46. doi: 10.1016/j.neuroscience.2015.03.011 25783477 PMC4527882

[206] Reynolds AD Stone DK Hutter JAL Benner EJ Mosley RL Gendelman HE. Regulatory T Cells Attenuate Th17 Cell-Mediated Nigrostriatal Dopaminergic Neurodegeneration in a Model of Parkinson’s Disease. J Immunol (2010) 184(5):2261–71. doi: 10.4049/jimmunol.0901852 PMC2824790 20118279

[207] Liu Z Huang Y Cao B-B Qiu Y-H Peng Y-P. Th17 Cells Induce Dopaminergic Neuronal Death *via* LFA-1/ICAM-1 Interaction in a Mouse Model of Parkinson’s Disease. Mol Neurobiol (2017) 54(10):7762–76. doi: 10.1007/s12035-016-0249-9 27844285

[208] Martin HL Santoro M Mustafa S Riedel G Forrester JV Teismann P. Evidence for a Role of Adaptive Immune Response in the Disease Pathogenesis of the MPTP Mouse Model of Parkinson’s Disease. Glia (2015) 64(3):386–95. doi: 10.1002/glia.22935 PMC4855685 26511587

[209] Luchtman DW Shao D Song C. Behavior, Neurotransmitters and Inflammation in Three Regimens of the MPTP Mouse Model of Parkinson’s Disease. Physiol Behav (2009) 98(1–2):130–8. doi: 10.1016/j.physbeh.2009.04.021 19410592

[210] Heuer A Smith GA Dunnett SB. Comparison of 6-Hydroxydopamine Lesions of the Substantia Nigra and the Medial Forebrain Bundle on a Lateralised Choice Reaction Time Task in Mice. Eur J Neurosci (2012) 37(2):294–302. doi: 10.1111/ejn.12036 23113688

[211] Ungerstedt U Arbuthnott GW. Quantitative Recording of Rotational Behavior in Rats After 6-Hydroxy-Dopamine Lesions of the Nigrostriatal Dopamine System. Brain Res (1970) 24(3):485–93. doi: 10.1016/0006-8993(70)90187-3 5494536

[212] Zigmond MJ Hastings TG Abercrombie ED. Neurochemical Responses to 6-Hydroxydopamine and L-Dopa Therapy: Implications for Parkinson’s Disease. Ann Ny Acad Sci (1992) 648(1 Neurotoxins a):71–86. doi: 10.1111/j.1749-6632.1992.tb24525.x 1637074

[213] Kupsch A Schmidt W Gizatullina Z Debska-Vielhaber G Voges J Striggow F. 6-Hydroxydopamine Impairs Mitochondrial Function in the Rat Model of Parkinson’s Disease: Respirometric, Histological, and Behavioral Analyses. J Neural Transm (2014) 121(10):1245–57. doi: 10.1007/s00702-014-1185-3 24627045

[214] Wheeler CJ Seksenyan A Koronyo Y Rentsendorj A Sarayba D Wu H. T-Lymphocyte Deficiency Exacerbates Behavioral Deficits in the 6-OHDA Unilateral Lesion Rat Model for Parkinson’s Disease. J Neurol Neurophysiol (2014) 05(03):209. doi: 10.4172/2155-9562.1000209 PMC4207300 25346865

[215] Koprich JB Johnston TH Reyes MG Sun X Brotchie JM. Expression of Human A53T Alpha-Synuclein in the Rat Substantia Nigra Using a Novel AAV1/2 Vector Produces a Rapidly Evolving Pathology With Protein Aggregation, Dystrophic Neurite Architecture and Nigrostriatal Degeneration With Potential to Model the Pathology of Parkinson’s Disease. Mol Neurodegener (2010) 5(1):43. doi: 10.1186/1750-1326-5-43 21029459 PMC2984491

[216] Theodore S Cao S McLean PJ Standaert DG. Targeted Overexpression of Human α-Synuclein Triggers Microglial Activation and an Adaptive Immune Response in a Mouse Model of Parkinson Disease. J Neuropathol Exp Neurol (2008) 67(12):1149–58. doi: 10.1097/NEN.0b013e31818e5e99 PMC2753200 19018246

[217] Williams GP Schonhoff AM Jurkuvenaite A Gallups NJ Standaert DG Harms AS. CD4 T Cells Mediate Brain Inflammation and Neurodegeneration in a Mouse Model of Parkinson Disease. Brain (2021) 144(7):2047–59. doi: 10.1093/brain/awab103 PMC8370411 33704423

[218] Harms AS Delic V Thome AD Bryant N Liu Z Chandra S. α-Synuclein Fibrils Recruit Peripheral Immune Cells in the Rat Brain Prior to Neurodegeneration. Acta Neuropathol Commun (2017) 5(1):85. doi: 10.1186/s40478-017-0494-9 29162163 PMC5698965

[219] Kim S Kwon S-H Kam T-I Panicker N Karuppagounder SS Lee S. Transneuronal Propagation of Pathologic α-Synuclein From the Gut to the Brain Models Parkinson’s Disease. Neuron (2019) 103(4):627–41.e7. doi: 10.1016/j.neuron.2019.05.035 31255487 PMC6706297

[220] Ramos JMP Iribarren P Bousset L Melki R Baekelandt V Perren AVd. Peripheral Inflammation Regulates CNS Immune Surveillance Through the Recruitment of Inflammatory Monocytes Upon Systemic α-Synuclein Administration. Front Immunol (2019) 10:80. doi: 10.3389/fimmu.2019.00080 30761145 PMC6361759

[221] Bonifati V Rizzu P van Baren MJ Schaap O Breedveld GJ Krieger E. Mutations in the DJ-1 Gene Associated With Autosomal Recessive Early-Onset Parkinsonism. Science (2002) 299(5604):256–9. doi: 10.1126/science.1077209 12446870

[222] Guo M. PINK1/PARKIN And Mitochondrial Dynamics In Neurodegeneration. Free Radical Bio Med (2017) 112:16. doi: 10.1016/j.freeradbiomed.2017.10.369

[223] Kitada T Asakawa S Hattori N Matsumine H Yamamura Y Minoshima S. Mutations in the Parkin Gene Cause Autosomal Recessive Juvenile Parkinsonism. Nature (1998) 392(6676):605–8. doi: 10.1038/33416 9560156

[224] Valente EM Abou-Sleiman PM Caputo V Muqit MMK Harvey K Gispert S. Hereditary Early-Onset Parkinson’s Disease Caused by Mutations in PINK1. Science (2004) 304(5674):1158–60. doi: 10.1126/science.1096284 15087508

[225] Weindel CG Bell SL Vail KJ West KO Patrick KL Watson RO. LRRK2 Maintains Mitochondrial Homeostasis and Regulates Innate Immune Responses to *Mycobacterium tuberculosis* . ELife (2020) 9:e51071. doi: 10.7554/eLife.51071 32057291 PMC7159881

[226] Aubin N Curet O Deffois A Carter C. Aspirin and Salicylate Protect Against MPTP-Induced Dopamine Depletion in Mice. J Neurochem (1998) 71(4):1635–42. doi: 10.1046/j.1471-4159.1998.71041635.x 9751197

[227] Ren L Yi J Yang J Li P Cheng X Mao P. Nonsteroidal Anti-Inflammatory Drugs Use and Risk of Parkinson Disease. Medicine (2018) 97(37):e12172. doi: 10.1097/MD.0000000000012172 30212946 PMC6155958

[228] Teismann P Tieu K Choi D-K Wu D-C Naini A Hunot S. Cyclooxygenase-2 is Instrumental in Parkinson’s Disease Neurodegeneration. Proc Natl Acad Sci (2003) 100(9):5473–8. doi: 10.1073/pnas.0837397100 PMC154369 12702778

[229] Feng Z-H Wang T-G Li D-D Fung P Wilson BC Liu B. Cyclooxygenase-2-Deficient Mice are Resistant to 1-Methyl-4-Phenyl1, 2, 3, 6-Tetrahydropyridine-Induced Damage of Dopaminergic Neurons in the Substantia Nigra. Neurosci Lett (2002) 329(3):354–8. doi: 10.1016/S0304-3940(02)00704-8 12183047

[230] Yamagata K Andreasson KI Kaufmann WE Barnes CA Worley PF. Expression of a Mitogen-Inducible Cyclooxygenase in Brain Neurons: Regulation by Synaptic Activity and Glucocorticoids. Neuron (1993) 11(2):371–86. doi: 10.1016/0896-6273(93)90192-T 8352945

[231] Andreasson K. Emerging Roles of PGE2 Receptors in Models of Neurological Disease. Prostag Oth Lipid M (2010) 91(3–4):104–12. doi: 10.1016/j.prostaglandins.2009.04.003 PMC2846228 19808012

[232] Caughey GE Cleland LG Penglis PS Gamble JR James MJ. Roles of Cyclooxygenase (COX)-1 and COX-2 in Prostanoid Production by Human Endothelial Cells: Selective Up-Regulation of Prostacyclin Synthesis by COX-2. J Immunol (2001) 167(5):2831–8. doi: 10.4049/jimmunol.167.5.2831 11509629

[233] Moncada S. Prostacyclin and Arterial Wall Biology. Arterioscler Off J Am Hear Assoc Inc (1982) 2(3):193–207. doi: 10.1161/01.ATV.2.3.193 6284099

[234] Jin J Shie F-S Liu J Wang Y Davis J Schantz AM. Prostaglandin E2 Receptor Subtype 2 (EP2) Regulates Microglial Activation and Associated Neurotoxicity Induced by Aggregated α-Synuclein. J Neuroinflamm (2007) 4(1):2. doi: 10.1186/1742-2094-4-2 PMC1766347 17204153

[235] Carrasco E Werner P Casper D. Prostaglandin Receptor EP2 Protects Dopaminergic Neurons Against 6-OHDA-Mediated Low Oxidative Stress. Neurosci Lett (2008) 441(1):44–9. doi: 10.1016/j.neulet.2008.05.111 PMC2570167 18597941

[236] Davies NM Wright MR Jamali F. Antiinflammatory Drug-Induced Small Intestinal Permeability: The Rat Is a Suitable Model. Pharmaceut Res (1994) 11(11):1652–6. doi: 10.1023/A:1018978308752 7870685

[237] Bjarnason I Williams P Smethurst P Peters TJ Levi AJ. Effect of non-Steroidal Anti-Inflammatory Drugs and Prostaglandins on the Permeability of the Human Small Intestine. Gut (1986) 27(11):1292–7. doi: 10.1136/gut.27.11.1292 PMC1434083 3466837

[238] Roy J Galano J Durand T Guennec JL Lee JC. Physiological Role of Reactive Oxygen Species as Promoters of Natural Defenses. FASEB J (2017) 31(9):3729–45. doi: 10.1096/fj.201700170R 28592639

[239] Lingappan K. NF-κb in Oxidative Stress. Curr Opin Toxicol (2018) 7:81–6. doi: 10.1016/j.cotox.2017.11.002 PMC5978768 29862377

[240] Groemping Y Rittinger K. Activation and Assembly of the NADPH Oxidase: A Structural Perspective. Biochem J (2005) 386(3):401–16. doi: 10.1042/BJ20041835 PMC1134858 15588255

[241] Rodriguez-Pallares J Rey P Parga JA Muñoz A Guerra MJ Labandeira-Garcia JL. Brain Angiotensin Enhances Dopaminergic Cell Death *via* Microglial Activation and NADPH-Derived ROS. Neurobiol Dis (2008) 31(1):58–73. doi: 10.1016/j.nbd.2008.03.003 18499466

[242] Wu D-C Teismann P Tieu K Vila M Jackson-Lewis V Ischiropoulos H. NADPH Oxidase Mediates Oxidative Stress in the 1-Methyl-4-Phenyl-1,2,3,6-Tetrahydropyridine Model of Parkinson’s Disease. Proc Natl Acad Sci (2003) 100(10):6145–50. doi: 10.1073/pnas.0937239100 PMC156340 12721370

[243] Parga JA Rodriguez-Perez AI Garcia-Garrote M Rodriguez-Pallares J Labandeira-Garcia JL. Angiotensin II Induces Oxidative Stress and Upregulates Neuroprotective Signaling From the NRF2 and KLF9 Pathway in Dopaminergic Cells. Free Radic Biol Med (2018) 129:394–406. doi: 10.1016/j.freeradbiomed.2018.10.409 30315936

[244] Belarbi K Cuvelier E Destée A Gressier B Chartier-Harlin M-C. NADPH Oxidases in Parkinson’s Disease: A Systematic Review. Mol Neurodegener (2017) 12(1):84. doi: 10.1186/s13024-017-0225-5 29132391 PMC5683583

[245] Tu D Gao Y Yang R Guan T Hong J-S Gao H-M. The Pentose Phosphate Pathway Regulates Chronic Neuroinflammation and Dopaminergic Neurodegeneration. J Neuroinflamm (2019) 16(1):255. doi: 10.1186/s12974-019-1659-1 PMC6896486 31805953

[246] Firbank MJ Yarnall AJ Lawson RA Duncan GW Khoo TK Petrides GS. Cerebral Glucose Metabolism and Cognition in Newly Diagnosed Parkinson’s Disease: ICICLE-PD Study. J Neurol Neurosurg Psychiatry (2016) 88(4):310–6. doi: 10.1136/jnnp-2016-313918 28315844

[247] Marques A Dutheil F Durand E Rieu I Mulliez A Fantini ML. Glucose Dysregulation in Parkinson’s Disease: Too Much Glucose or Not Enough Insulin? Parkinsonism Relat D (2018) 55:122–7. doi: 10.1016/j.parkreldis.2018.05.026 29866628

[248] Pagano G Polychronis S Wilson H Giordano B Ferrara N Niccolini F. Diabetes Mellitus and Parkinson Disease. Neurology (2018) 90(19):e1654–62. doi: 10.1212/WNL.0000000000005475 29626177

[249] Stanton RC. Glucose-6-Phosphate Dehydrogenase, NADPH, and Cell Survival. IUBMB Life (2012) 64(5):362–9. doi: 10.1002/iub.1017 PMC3325335 22431005

[250] Dunn L Allen GFG Mamais A Ling H Li A Duberley KE. Dysregulation of Glucose Metabolism Is an Early Event in Sporadic Parkinson’s Disease. Neurobiol Aging (2014) 35(5):1111–5. doi: 10.1016/j.neurobiolaging.2013.11.001 PMC3969149 24300239

[251] Braak H Sastre M Bohl JRE de Vos RAI Tredici KD. Parkinson’s Disease: Lesions in Dorsal Horn Layer I, Involvement of Parasympathetic and Sympathetic Pre- and Postganglionic Neurons. Acta Neuropathol (2007) 113(4):421–9. doi: 10.1007/s00401-007-0193-x 17294202

[252] Chinta SJ Andersen JK. Reversible Inhibition of Mitochondrial Complex I Activity Following Chronic Dopaminergic Glutathione Depletion *In Vitro*: Implications for Parkinson’s Disease. Free Radical Bio Med (2006) 41(9):1442–8. doi: 10.1016/j.freeradbiomed.2006.08.002 17023271

[253] Mischley LK Lau RC Shankland EG Wilbur TK Padowski JM. Phase IIb Study of Intranasal Glutathione in Parkinson’s Disease. J Park Dis (2017) 7(2):289–99. doi: 10.3233/JPD-161040 PMC5438472 28436395

[254] Sian J Dexter DT Lees AJ Daniel S Agid Y Javoy-Agid F. Alterations in Glutathione Levels in Parkinson’s Disease and Other Neurodegenerative Disorders Affecting Basal Ganglia. Ann Neurol (1994) 36(3):348–55. doi: 10.1002/ana.410360305 8080242

[255] Grammatopoulos TN Jones SM Ahmadi FA Hoover BR Snell LD Skoch J. Angiotensin Type 1 Receptor Antagonist Losartan, Reduces MPTP-Induced Degeneration of Dopaminergic Neurons in Substantia Nigra. Mol Neurodegener (2007) 2(1):1. doi: 10.1186/1750-1326-2-1 17224059 PMC1783655

[256] Laghlam D Jozwiak M Nguyen LS. Renin-Angiotensin-Aldosterone System and Immunomodulation: A State-Of-the-Art Review. Cells (2021) 10(7):1767. doi: 10.3390/cells10071767 34359936 PMC8303450

[257] te Riet L van Esch JHM Roks AJM van den Meiracker AH Danser AHJ. Hypertension. Circ Res (2015) 116(6):960–75. doi: 10.1161/CIRCRESAHA.116.303587 25767283

[258] Rey P Lopez-Real A Sanchez-Iglesias S Muñoz A Soto-Otero R Labandeira-Garcia JL. Angiotensin Type-1-Receptor Antagonists Reduce 6-Hydroxydopamine Toxicity for Dopaminergic Neurons. Neurobiol Aging (2007) 28(4):555–67. doi: 10.1016/j.neurobiolaging.2006.02.018 16621167

[259] Joglar B Rodriguez-Pallares J Rodriguez-Perez AI Rey P Guerra MJ Labandeira-Garcia JL. The Inflammatory Response in the MPTP Model of Parkinson’s Disease is Mediated by Brain Angiotensin: Relevance to Progression of the Disease. J Neurochem (2009) 109(2):656–69. doi: 10.1111/j.1471-4159.2009.05999.x 19245663

[260] Chabrashvili T Kitiyakara C Blau J Karber A Aslam S Welch WJ. Effects of ANG II Type 1 and 2 Receptors on Oxidative Stress, Renal NADPH Oxidase, and SOD Expression. Am J Physiology-regulatory Integr Comp Physiol (2003) 285(1):R117–24. doi: 10.1152/ajpregu.00476.2002 12609817

[261] Wright JW Kawas LH Harding JW. A Role for the Brain RAS in Alzheimer’s and Parkinson’s Diseases. Front Endocrinol (2013) 4:158. doi: 10.3389/fendo.2013.00158 PMC3829467 24298267

[262] Muñoz A Rey P Guerra MJ Mendez-Alvarez E Soto-Otero R Labandeira-Garcia JL. Reduction of Dopaminergic Degeneration and Oxidative Stress by Inhibition of Angiotensin Converting Enzyme in a MPTP Model of Parkinsonism. Neuropharmacology (2006) 51(1):112–20. doi: 10.1016/j.neuropharm.2006.03.004 16678218

[263] Dalmay F Pesteil F Nisse-Durgeat S Fournier A Achard JM. Angiotensin IV Decreases Acute Stroke Mortality in the Gerbil. Am J Hypertens (2001) 14(11):A56. doi: 10.1016/S0895-7061(01)01592-8

[264] Kramár EA Harding JW Wright JW. Angiotensin II- and IV-Induced Changes in Cerebral Blood Flow. Regul Peptides (1997) 68(2):131–8. doi: 10.1016/S0167-0115(96)02116-7 9110385

[265] Näveri L Strömberg C Saavedra JM. Angiotensin IV Reverses the Acute Cerebral Blood Flow Reduction After Experimental Subarachnoid Hemorrhage in the Rat. J Cereb Blood Flow Metab (1994) 14(6):1096–9. doi: 10.1038/jcbfm.1994.143 7523429

[266] Benoist CC Wright JW Zhu M Appleyard SM Wayman GA Harding JW. Facilitation of Hippocampal Synaptogenesis and Spatial Memory by C-Terminal Truncated Nle 1 -Angiotensin IV Analogs. J Pharmacol Exp Ther (2011) 339(1):35–44. doi: 10.1124/jpet.111.182220 21719467 PMC3186286

[267] Zawada WM Mrak RE Biedermann J Palmer QD Gentleman SM Aboud O. Loss of Angiotensin II Receptor Expression in Dopamine Neurons in Parkinson’s Disease Correlates With Pathological Progression and Is Accompanied by Increases in Nox4- and 8-OH Guanosine-Related Nucleic Acid Oxidation and Caspase-3 Activation. Acta Neuropathol Commun (2015) 3(1):9. doi: 10.1186/s40478-015-0189-z 25645462 PMC4359535

[268] El-Arif G Farhat A Khazaal S Annweiler C Kovacic H Wu Y. The Renin-Angiotensin System: A Key Role in SARS-CoV-2-Induced COVID-19. Mol Basel Switz (2021) 26(22):6945. doi: 10.3390/molecules26226945 PMC8622307 34834033

